# Systems-Level Transcriptomic and Cellular Architecture of the Human Striatum in Schizophrenia and Bipolar Psychosis

**DOI:** 10.3390/biology15191714

**Published:** 2026-09-26

**Authors:** Wei-Hung Chan, Shih-Ming Huang, Hui-Chen Su, Yi-Lin Chiu

**Affiliations:** 1 Department of Anesthesiology, Tri-Service General Hospital, National Defense Medical University, Taipei City 114202, Taiwan; whcken@gmail.com; 2 Department of Anesthesiology, School of Medicine, College of Medicine, National Defense Medical University, Taipei City 114201, Taiwan; 3 Graduate Institute of Biochemistry, College of Biomedical Sciences, National Defense Medical University, Taipei City 114201, Taiwan; 4 Department of Pharmacy, Chi-Mei Medical Center, Tainan City 710402, Taiwan

**Keywords:** schizophrenia, bipolar disorder with psychosis, striatum, molecular neurobiology, transcriptomics, neuroimmune signaling, cell-type deconvolution, oligodendrocyte lineage

## Abstract

Psychosis involves striatal circuit dysfunction, but the regional molecular and cellular changes remain unclear. We re-analyzed 215 postmortem RNA-seq samples (72 donors: 36 controls, 28 schizophrenia, 8 bipolar disorder with psychosis) from the nucleus accumbens, caudate and putamen, asking whether diagnosis-associated transcriptional changes are region-specific and whether they reflect cellular composition or cell-intrinsic state. Region-specific limma-voom models identified 0, 141 and 2152 schizophrenia-associated genes (FDR < 0.10) in the nucleus accumbens, caudate and putamen; the small bipolar-psychosis group yielded one gene, with limited power to detect additional associations. Correlation-aware camera tests supported immune–inflammatory Hallmark programs in the caudate and putamen, whereas nucleus accumbens signals were not robust to sequencing-run adjustment. Leukocyte-reference deconvolution was uninformative for brain parenchyma. Across four deconvolution estimators and two single-nucleus references, including an independent eight-donor striatal reference, putamen samples from donors with schizophrenia showed a consistently lower oligodendrocyte-lineage reference weight, whereas attribution to oligodendrocyte precursor cells specifically was method-dependent. Exploratory directional signatures (dopamine–adenosine, glutamate–calcium, Activin–SMAD, APP processing) remained supported in the caudate and putamen after direction-selection permutation conditional on fixed gene membership and were driven mainly by schizophrenia. The results characterize a dorsal–striatal, schizophrenia-associated transcriptional and oligodendroglial signal and provide prioritized hypotheses for independent validation.

## Simple Summary

Psychosis, a loss of contact with reality that occurs in schizophrenia and in some forms of bipolar disorder, is linked to a deep brain structure called the striatum. Using publicly available gene-activity data from three striatal regions of 72 donors, we asked whether the molecular changes seen in schizophrenia and in bipolar psychosis differ between regions and whether these changes reflect altered proportions of brain cell types or altered activity within cells. In schizophrenia, gene-activity changes were concentrated in the putamen and, to a lesser degree, the caudate, and inflammation-related gene programs were prominent in both. Estimates of cell composition consistently pointed to a lower share of oligodendrocyte-lineage cells, which insulate nerve fibers, in the schizophrenia putamen, whereas immune-cell references were not informative for brain tissue. The bipolar psychosis group was small, so its results are inconclusive rather than negative. All findings come from one dataset and are exploratory; they provide testable hypotheses about where, and in which cell types, psychosis-related molecular changes occur in the human striatum, rather than proof of mechanism.

## 1. Introduction

Psychosis is increasingly conceptualized as a transdiagnostic clinical dimension rather than a syndrome confined to one diagnostic category. Psychotic symptoms occur across schizophrenia-spectrum disorders, bipolar disorder, depressive disorders, and help-seeking youth populations, and dimensional models often capture shared symptom structure better than strict categorical boundaries [1,2,3,4,5]. Biological evidence is less mature: shared symptom structure and risk stratification are better established than replicated biomarkers or treatment-guiding molecular mechanisms, although cross-disorder transcriptomic studies support partially shared molecular pathology [3,6,7,8].

The striatum is a plausible anatomical substrate for this gap because psychosis is consistently linked to cortico-striatal and cortico-striato-thalamic circuit dysfunction. Functional dysconnectivity has been reported across psychosis-risk states, first-episode psychosis, schizophrenia, psychotic bipolar disorder, and subclinical psychosis-like experiences [9,10,11,12,13,14]. These abnormalities are prominent in dorsal associative circuits involving the caudate, prefrontal cortex, and thalamus, but they also extend to the limbic, salience, cerebellar, and dopaminergic systems. Their associations with cognition, symptom severity, and antipsychotic response suggest that striatal molecular changes may provide biological context for clinically relevant behavioral and circuit phenotypes, although transcriptomic data alone cannot establish that link [12,15,16].

Striatal subregions cannot be treated as a single molecular compartment. The nucleus accumbens, caudate, and putamen differ in cellular composition, connectivity, baseline transcriptional organization, and functional specialization. The putamen is repeatedly implicated in psychosis-related connectivity and symptom dimensions [14,17,18], while postmortem evidence links major psychiatric disorders to oligodendrocyte pathology in the anterior putamen [19]. Region-resolved human transcriptomic studies further show that psychosis-associated expression varies across striatal subregions and interacts with diurnal, sex-related, and regional regulatory programs [20,21,22,23].

A systems-level analysis is needed to separate three sources of bulk-tissue signal: coordinated molecular programs, shifts in cellular composition, and cell-state changes within relatively stable populations. Hallmark gene sets reduce redundancy among biological programs [24], whereas reference-based deconvolution can test whether bulk expression differences track leukocyte or brain-resident cellular profiles [25,26,27,28]. Single-nucleus profiling across the human reward circuitry further demonstrates that relevant cell types carry region-specific transcriptional signatures [29]. These layers are complementary rather than interchangeable. A pathway-level inflammatory signal, for example, need not imply increased immune-cell abundance, and a reference-derived composition estimate does not demonstrate cell-intrinsic activation.

Genetic-regulatory integration provides a further axis of interpretation. Most psychiatric GWAS loci are noncoding, making brain eQTL and transcriptome-wide approaches useful for linking risk loci to candidate genes [30,31,32,33,34,35,36]. Yet gene-level eQTL and mapped-gene annotations remain prioritization evidence unless formal variant-level colocalization is performed. A coherent systems model should therefore distinguish transcriptomic state, inferred cellular context, and external genetic support rather than collapsing them into a single causal claim.

Here, we re-analyzed GSE202537 across the nucleus accumbens, caudate, and putamen to address one primary question: are schizophrenia- and bipolar-psychosis-associated striatal transcriptional changes region-specific, and do they reflect shifts in cellular composition or cell-intrinsic state? Three aims followed. Aim 1 estimated diagnosis-specific gene- and Hallmark-level changes within each region with an established RNA-seq framework (limma-voom) and correlation-aware gene-set tests. Aim 2 asked whether these changes were accompanied by composition shifts, using a tissue-matched human brain single-nucleus reference as the primary estimator and leukocyte- or cerebrospinal-fluid-based immune references only as sensitivity analyses. Aim 3 related the regional findings to prioritized molecular axes and to external genetic-regulatory evidence, treating within-dataset signature analyses as exploratory. Because the source dataset lacks granular symptom and behavioral measurements, behavioral relevance was inferred from established striatal circuit biology and was not tested directly.

## 2. Materials and Methods

### 2.1. Public Data Acquisition and Annotation

GSE202537 series-matrix metadata and the public read-count matrix for the NAc, caudate, and putamen were obtained from GEO-associated resources. Gene annotation used the Ensembl Homo sapiens GRCh38 release 110 GTF. Hallmark gene sets were obtained from the MSigDB Hallmark 2020 Enrichr library [24], and the Elsevier Pathway Collection was retained for the secondary pathway analysis. GTEx v8 independent cis-eQTL records and GWAS Catalog mapped-gene associations were queried through their public APIs. Pure-brain reference profiles were obtained from the HPA single-nucleus brain cluster-type tables derived from Siletti et al. [27]. The CNS-adjacent reference was the CELLxGENE Discover dataset entitled ‘Immune Response mechanisms in the cerebrospinal fluid in leptomeningeal disease at single cell resolution’ (dataset version 0d240f09-c7d4-4420-89ca-376d19663777; collection 573e2e06-8af0-4d96-bfdd-7d64a4bb9c21), associated with Nieto et al. [37]. Original tissue procurement, donor recruitment, informed consent, sequencing-library preparation, and institutional ethics approval were performed by the original investigators and are described in the source publications and GEO records [20,37].

### 2.2. Clinical Metadata Processing and Quality Control

Sample-level characteristics were parsed from the GSE202537 series matrix and harmonized by GEO accession. Diagnostic groups were standardized as control, schizophrenia, and bipolar disorder with psychosis; brain regions were encoded as NAc, caudate, and putamen. Donors were deduplicated by the source patient identifier for clinical summaries, whereas sample-level records were retained for region counts and transcriptomic analyses. Corrected time of death was represented on a 24 h scale and transformed into sine and cosine terms. Donor-level age, postmortem interval, RNA integrity number, brain pH, and corrected time of death were compared by Kruskal–Wallis tests; sex and other available categorical variables were compared by chi-square tests. Boxplots show median and interquartile range with individual observations. Sequencing run (flow-cell identifier) was recorded for every sample; runs were nested within region, and their alignment with diagnosis within each region was tabulated (Supplementary Data S1).

Read counts were indexed by Ensembl identifiers, mapped to gene symbols using the GRCh38 release 110 annotation, and summed when multiple identifiers mapped to one symbol. Genes were retained when raw counts were at least 10 in at least 10% of samples. Counts were converted to counts per million and transformed as log_2_(CPM + 1). Quality-control metrics included library depth and detected-gene count. Principal-component analysis was performed on processed expression values. Associations between principal components and continuous covariates used Spearman correlation, whereas categorical covariates used Kruskal–Wallis tests.

### 2.3. Diagnosis-Specific Gene Models (Limma-Voom) and Hallmark Gene-Set Tests (Camera)

Gene-level models were fitted separately within the NAc, caudate, and putamen with limma-voom [38,39]. Filtered symbol-level counts were normalized by the trimmed mean of M values (edgeR [40]), converted to log_2_-CPM with precision weights by voom, and fitted by weighted least squares with empirical Bayes moderation of the residual variances. The design matrix contained simultaneous indicator variables for schizophrenia and bipolar disorder with psychosis, with control as the reference, plus age, sex, postmortem interval, RNA integrity number, brain pH, sequencing depth, and sine/cosine-transformed corrected time of death; continuous covariates were standardized within each regional model. Coefficients are reported as limma log_2_ fold changes with moderated t statistics, and Benjamini–Hochberg FDR correction was performed within each diagnosis-region family. An ordinary-least-squares model on log_2_(CPM + 1), which does not model the count mean-variance relationship, was fitted with the same design for comparison only (Supplementary Figure S5). Sensitivity analyses added sequencing run as a fixed effect, modeled run as a random block with duplicateCorrelation (two-pass estimation) [41], or added RUVr residual factors (k = 1–3) [42] or svaseq surrogate variables [43] to the design; variants were compared with the primary model by rank correlation of t statistics, sign concordance of FDR-supported genes, and overlap of FDR-supported sets. Storey’s π_0_ [44] and *p*-value histograms were used to describe the regional gradient. Genome-wide schizophrenia–bipolar concordance and regional-gradient summaries were calculated from these same primary R/limma-voom estimates. Bipolar-arm power was approximated using the two-sided noncentral t distribution at alpha = 0.05, with the fitted moderated standard errors and degrees of freedom; schizophrenia effect estimates were used as hypothetical effect sizes. The approximate 80% minimum detectable effect was calculated as the sum of the 97.5th and 80th percentiles of the corresponding central t distribution, multiplied by the gene-specific standard error. These plug-in calculations describe sensitivity under the fitted model and do not provide independent evidence of an effect.

The 50 Hallmark gene sets were tested with camera [45], a competitive test that accounts for inter-gene correlation, applied to the voom objects and design matrices with the default inter-gene correlation of 0.01 and, as a sensitivity analysis, with correlations estimated from the residuals; fry provided a self-contained directional test. Benjamini–Hochberg correction was applied across 50 sets within each diagnosis-region family. The sets were grouped a priori into seven systems classes for visualization: immune–inflammatory; stress and cell death; metabolism and mitochondria; cell cycle and genome; developmental signaling; growth and kinase signaling; and hormone and tissue remodeling. A gene-sampling permutation test (mean gene-level t statistic against 2000 same-size random sets; random seed 17), which does not preserve inter-gene correlation, is reported only for comparison (Supplementary Figure S4).

### 2.4. IOBR-CIBERSORT LM22 Immune Deconvolution (Sensitivity Analysis)

Gene-level CPM values were supplied to the IOBR implementation of CIBERSORT [25,26] with the LM22 signature matrix, RNA-seq mode, 100 permutations, relative mode, and random seed 20260706. The resulting 22 fractions were analyzed in schizophrenia and control samples within each brain region. Group means were used only for stacked-composition summaries. Sample-level fractions were compared by two-sided Mann–Whitney U tests, and Benjamini–Hochberg correction was applied both within each region and across all region–cell-type comparisons. Because LM22 is a leukocyte reference, these estimates were interpreted as relative transcriptomic fractions rather than direct counts of cells in brain tissue. Because LM22 was developed for leukocyte mixtures rather than brain tissue, these estimates were treated as a sensitivity analysis and were not interpreted as evidence for or against immune-cell infiltration.

### 2.5. CD4 Memory State Analysis (Sensitivity Analysis)

Resting and activated CD4 memory fractions were extracted from the LM22 CIBERSORT output. The activation balance was defined as log_2_[(activated + 0.001)/(resting + 0.001)]. To construct independent expression-based representations, LM22 genes were ranked by specificity for each CD4 memory state relative to the maximum expression in other LM22 cell types, and the top 30 genes per state were retained. Marker scores were calculated as the mean z-scored expression of available marker genes. Projection scores were the Spearman correlations between each bulk-sample expression profile and the corresponding LM22 target profile; marker and projection balances were calculated as activated minus resting. Each metric was tested by region-specific OLS with schizophrenia status, age, sex, postmortem interval, RNA integrity number, brain pH, sequencing depth, and sine/cosine time-of-death terms. FDR correction was applied within region. Immune Hallmark scores were calculated as mean z-scored expression of available set members, correlated with the CIBERSORT activation balance by Spearman correlation, and corrected within region.

### 2.6. Human Brain Single-Nucleus Reference Deconvolution and Alternative Estimators

HPA single-nucleus expression was pivoted to gene-by-cluster profiles and collapsed into seven classes: MSN, interneuron, astrocyte, oligodendrocyte, OPC, microglia, and vascular. For each class, expression was averaged across mapped HPA/Siletti clusters. Marker specificity was defined as log_2_[(mean class nCPM + 1)/(maximum nCPM among other clusters + 1)]. Genes with class nCPM ≥ 1 were ranked by specificity and expression, and up to 80 markers per class were retained; 70–80 markers per class overlapped GSE202537. Reference profiles and bulk CPM values were transformed as log_2_(value + 1). Non-negative least-squares regression estimated one coefficient per class for every bulk sample; coefficients were normalized to sum to one. These values are referred to as reference weights, not cell fractions. Schizophrenia effects were tested within each region by OLS on arcsine-square-root-transformed weights, adjusting for age, sex, postmortem interval, RNA integrity number, brain pH, library size, and cyclic time of death. FDR was calculated within region and globally across all class–region tests.

Three alternative estimators were applied to the same bulk data to assess reference and scale dependence. (i) Linear-scale NNLS was fitted after dividing each marker gene in both the reference and the bulk matrix by the reference row mean. (ii) Nu-support-vector regression was run with the IOBR implementation of CIBERSORT [25,26] using the 523-gene HPA/Siletti signature matrix (100 permutations, relative mode, no quantile normalization). (iii) MuSiC [28] was run with an independent multi-donor human NAc single-nucleus reference (Tran et al. [29]; SCE_NAc-n8, 19,892 nuclei after excluding doublet and low-transcript clusters, eight donors, 16,181 genes shared with the bulk matrix), with cluster labels collapsed into MSN, interneuron, astrocyte, oligodendrocyte, OPC, and microglia and donors as reference subjects. Oligodendrocyte-lineage weight was defined as the sum of the OPC and oligodendrocyte estimates. Estimator behavior was benchmarked in 300 simulated mixtures generated from HPA/Siletti cluster profiles with Dirichlet-distributed proportions (OPC 0.5–10%) and Poisson sampling at 3 × 10^7^ reads, and by leave-one-cluster-out re-derivation of the marker reference. All estimates were tested with the covariate-adjusted arcsine model, with and without sequencing run as a fixed effect, and cross-method agreement was quantified by Spearman correlation of sample-level estimates.

### 2.7. CELLxGENE CSF MuSiC Sensitivity Analysis

The CELLxGENE CSF reference [37] was restricted to immune cells by excluding non-immune, malignant, and tumor annotations. Level-2 cell states with at least 100 cells and at least three donors were retained. To limit domination by abundant states, at most 250 cells per state were sampled with random seed 20260707. The resulting general reference contained 6684 cells from 17 donors, 27 immune states, and 16,444 genes shared with the bulk matrix. MuSiC [28] was fitted to CPM-scale bulk expression with cell-state labels as clusters, donor identifiers as reference subjects, internal normalization disabled, and random seed 20260707. A second model was conditioned on seven CD4-related states: naive CD4, central-memory CD4, regulatory CD4, Th17, CD4 helper, CD4 interferon response, and proliferative T cells. This conditioned reference contained 1750 cells from 17 donors. Weighted MuSiC proportions were tested by the same region-specific covariate-adjusted OLS design used for HPA/Siletti weights after arcsine-square-root transformation. Benjamini–Hochberg FDR was calculated within region and globally.

### 2.8. Elsevier Pathway, Candidate-Gene, and Signature Analyses

Elsevier pathway sets were intersected with expressed genes and filtered to retain 5–500 members. For each sample, genes were ranked by expression, and a weighted rank-based score was calculated for every pathway and then standardized across samples. Region-specific pooled-psychosis effects combined schizophrenia and bipolar disorder with psychosis and adjusted for age, sex, postmortem interval, RNA integrity number, brain pH, sequencing depth, and cyclic time of death. Benjamini–Hochberg correction was applied within region. Candidate genes from selected neuropsychiatric pathways were tested by the same design.

Five directional signatures were assembled from candidate genes of the selected pathways. Gene membership was curated using pathway biology after data-dependent pathway screening and was held fixed during the permutation analysis: Activin–SMAD (INHBA, SMAD1, SMAD3, ACVR1C, SMURF1, GDF5, GDF11), dopamine–adenosine (TH, MAOB, ADK, ADORA1, ADORA2A, IL6), glutamate–calcium (GRM8, GNAO1, XBP1, SLC2A1, CACNA2D1, HSPA5, CACNA1C), immune-NF-kB (NFKBIA, CEBPB, CXCL8, PRKCZ, TIMP2, CCL2, IL1B), and APP processing (PSEN1, KAT5, ADAM17, APP, BACE1, ADAM10, PSEN2). Each gene’s direction (+1 or −1) was assigned from the sign of its pooled-psychosis coefficient in the region with the smallest *p* value; the directions were therefore data-informed rather than prespecified. Expression values were z-standardized across samples, multiplied by the assigned direction, and averaged within signature. Signature scores were tested with the pooled-psychosis regional model and, separately, with simultaneous schizophrenia and bipolar-psychosis indicators (95% confidence intervals and a Wald test of the difference). For 1000 donor-level permutations, psychosis labels were shuffled across donors and the same permuted donor label was applied across regions. Direction assignment, scoring, and regional testing were repeated in every permutation while signature gene membership was held fixed. The empirical *p* value was calculated as (1 + the number of permuted absolute t statistics at least as large as the observed absolute t statistic)/(1 + the number of permutations). Benjamini–Hochberg correction was applied across the five signatures within each region. These direction-selection-aware *p* values were conditional on the selected genes; neither the initial pathway screen nor candidate-gene selection was repeated. Cross-region transfer assigned directions in one region and tested the resulting scores in another; the shared donors precluded independent replication.

### 2.9. eQTL/GWAS Integration and Cell-Marker Cross-Reference

Signature genes were mapped to GTEx-compatible GENCODE identifiers through the GTEx Portal reference-gene endpoint. Independent cis-eQTL records were queried in GTEx v8 caudate, putamen, nucleus accumbens, cortex, and frontal cortex BA9. For each gene–tissue pair, record counts, minimum *p* value, and top variant were retained. GWAS Catalog REST API v2 was queried by mapped gene for schizophrenia, psychotic disorder, bipolar disorder, major depressive disorder, autism spectrum disorder, and ADHD. Association counts, minimum *p* values, top rs identifiers, and source-study metadata were recorded. Genes were classified as eQTL only, GWAS only, both, or neither. No colocalization was performed because harmonized variant-level summary statistics and linkage disequilibrium information were not supplied.

Curated marker panels represented D1 MSN, D2 MSN, interneuron, dopaminergic axon, astrocyte, oligodendrocyte, OPC, microglia, and vascular programs. Signature-marker overlap used one-sided Fisher exact tests with the expressed gene universe and Benjamini–Hochberg correction. Marker scores used the rank-based pathway-scoring procedure and the pooled-psychosis regional model. For the external cross-reference, 34 HPA/Siletti cluster types were collapsed into seven broad classes. The top 40 class-specific genes were selected by log_2_ specificity against the maximum other-class expression, rescored in the same bulk samples, and compared with internal marker-score effects by Spearman correlation; this comparison is within-dataset triangulation, not independent replication.

### 2.10. Visualization, Software, and Reproducibility

Python 3.9.13 (pandas 2.3.3, NumPy 2.0.2, SciPy 1.13.1, matplotlib 3.9.4, seaborn 0.13.2, Pillow 11.3.0) was used for data processing, the pathway, candidate-gene, signature, eQTL/GWAS and cell-marker workflows, the single-nucleus reference deconvolution, statistical summaries, and visualization; Python 3.11.15 (pandas 3.0.2, NumPy 2.4.4, SciPy 1.17.1, matplotlib 3.10.9, seaborn 0.13.2, Pillow 12.2.0) was used for the donor-level permutation analysis, the alternative non-negative least-squares estimators and mixture simulation, the regional-gradient diagnostics, and the rendering of Figures 1–3. R 4.5.2 with edgeR 4.8.2, limma 3.66.0, RUVSeq 1.44.0, sva 3.58.0, MuSiC 1.0.0, and IOBR 0.99.99 was used for TMM normalization, voom, camera, fry, duplicateCorrelation, RUVr, svaseq, CIBERSORT, and MuSiC; the complete session information is deposited with the code. All hypothesis tests were two-sided unless otherwise stated. Regional FDR < 0.10 was the prespecified discovery threshold; global FDR values are additionally reported for the reference-based composition analyses. Analysis outputs were retained as versioned, machine-readable tables. Executable code for all workflows, including the custom permutation, NNLS, marker-selection, directional-signature, and sensitivity analyses, is released in the public code repository listed in the Data Availability Statement.

### 2.11. Evidence-Grading Criteria

To make the weighting of evidence explicit, every principal finding was graded on four criteria (Table 1): (1) whether the statistic derives from an established framework with false-discovery control; (2) whether the reference or gene set is matched to striatal tissue; (3) whether an orthogonal method applied to the same data agrees; and (4) whether independent external data support the direction. Tier 1 findings meet criteria 1 and 3 and are robust to sequencing-run adjustment; tier 2 findings additionally meet criteria 2 and 4 but depend on reference-based estimation; tier 3 findings are within-dataset and data-informed and are reported as exploratory; annotations from external databases are prioritization evidence and are not graded. Where evidence layers conflict, a reference that is not matched to the tissue is treated as uninformative rather than as counter-evidence.

## 3. Results

### 3.1. Cohort Structure and Transcriptome Quality Support Region-Resolved Modeling

We first assessed whether diagnostic groups differed in sampling structure or major donor-level covariates. The cohort comprised 72 donors: 36 controls, 28 individuals with schizophrenia, and eight individuals with bipolar disorder with psychosis. Across 215 bulk RNA-seq samples, 71 were from the nucleus accumbens (NAc), 72 were from the caudate, and 72 were from the putamen. Control and schizophrenia donors were represented across all three regions; one bipolar psychosis donor lacked an NAc sample (Supplementary Figure S1A). The donor-level group counts are shown separately in Supplementary Figure S1B. Age (Supplementary Figure S1C), sex (Supplementary Figure S1D), postmortem interval (Supplementary Figure S1E), RNA integrity number (Supplementary Figure S1F), brain pH (Supplementary Figure S1G), and corrected time of death (Supplementary Figure S1H) did not differ significantly among diagnostic groups.

Expression quality control showed a median sequencing depth of 32.81 million reads (Supplementary Figure S2A) and a median of 32,358 detected genes per sample (Supplementary Figure S2B). Principal components 1 and 2 explained 24.8% and 13.1% of total variance, respectively (Supplementary Figure S2C). Samples separated primarily by brain region (Supplementary Figure S2D) rather than diagnosis (Supplementary Figure S2E), and sequencing run, sex, RNA integrity, count depth, and corrected time of death contributed to selected leading components (Supplementary Figure S2F). Sequencing runs were nested within region (10, 12, and 10 flow cells in NAc, caudate, and putamen, with no run shared between regions), so regional and run-related contributions to the between-region separation could not be fully distinguished. Within the NAc, run was also partly aligned with diagnosis (two runs contained 20 control samples only and two runs contained 11 schizophrenia samples only), a structure that motivated the run-sensitivity analyses reported below. These observations motivated region-specific models with explicit clinical and technical covariate adjustment.

### 3.2. Schizophrenia-Associated Transcriptomic Burden Increases from Ventral to Dorsal Striatum

To establish an unbiased systems-level starting point, we modeled schizophrenia and bipolar psychosis separately within each region across 20,810 expressed genes with limma-voom, using the same covariate set in all analyses (age, sex, postmortem interval, RNA integrity number, brain pH, sequencing depth, and cyclic time of death). Schizophrenia showed a pronounced dorsal striatal gradient: no gene reached FDR < 0.10 in the NAc, 141 genes did so in the caudate, and 2152 did so in the putamen (46 and 1036 at FDR < 0.05; Figure 1A). SCML1, the only NAc gene supported by the comparison ordinary-least-squares model, had FDR = 0.22 under limma-voom. Caudate signals included PMEPA1 (log_2_ fold change = 0.40, FDR = 0.0013), B4GALT5 (0.19), ASPHD2 (0.31), DOK5 (−0.33), CA12 (0.40), CHST15 (0.26), and INHBA (0.63, FDR = 0.004). The putamen showed the broadest signal, led by INHBA (0.81, FDR = 7 × 10^−6^), ETNK2 (−0.57, FDR = 4 × 10^−5^), NRSN1, VCPKMT, CALCB, NAA40, SLC7A2, and PXN-AS1. In bipolar psychosis, only CCBE1 in the caudate reached FDR < 0.10 (log_2_ fold change = 1.84, FDR = 0.074); the minimum FDR was 1.00 in the NAc and 0.56 in the putamen (Figure 1B). Among schizophrenia-supported genes, bipolar-psychosis effects were largely directionally concordant but attenuated (Supplementary Figure S3A). With eight donors and only one FDR-supported gene, the broader bipolar-psychosis pattern remained inconclusive: the median standard error of bipolar-psychosis coefficients was 1.5–1.7 times that of schizophrenia, and 77% (caudate) and 95% (putamen) of the schizophrenia-associated effect sizes fell below the effect detectable with 80% power in the bipolar-psychosis arm (Supplementary Figure S3B).

We next asked whether coordinated gene sets were detectable, using camera [45], a competitive gene-set test that accounts for inter-gene correlation, with the default correlation of 0.01. In schizophrenia, 13, 20, and 19 of the 50 Hallmark sets reached camera FDR < 0.10 in the NAc, caudate, and putamen (Figure 1C). The caudate had the strongest immune–inflammatory enrichment (TNFA/NF-kB and IL6/JAK/STAT3, FDR < 0.001; IL2/STAT5, FDR = 0.001; inflammatory response, 0.011; interferon-gamma response, 0.021; complement, 0.048), together with hypoxia, estrogen-response, and TGF-beta programs. The putamen combined IL6/JAK/STAT3 (FDR = 0.008), allograft rejection (0.009), and IL2/STAT5 (0.045) with borderline inflammatory-response, TNFA/NF-kB, and complement programs (FDR 0.053–0.059) and with protein-secretion, MYC-target, mTORC1, and unfolded-protein-response enrichment (Figure 1D). Bipolar psychosis retained nine camera-supported sets in the NAc, dominated by oxidative-phosphorylation, MYC-target, fatty-acid-metabolism, and mTORC1 programs, and one set each in the caudate and putamen. These counts are lower than those from the gene-sampling permutation test (18, 34, and 23 schizophrenia sets, Supplementary Figure S4A; 24, 7, and 2 bipolar sets, Supplementary Figure S4B), which does not preserve inter-gene correlation. When the correlation was instead estimated from the data (median 0.04–0.05 per set; Supplementary Figure S4D), only one caudate set remained, and the self-contained fry test supported 5 caudate and 14 putamen sets (Supplementary Figure S4C). Hallmark conclusions are therefore stated at the level supported by camera with the default correlation and are qualified accordingly.

The gene-level gradient was robust to the modeling framework and to sequencing-run adjustment (Supplementary Figure S5A). Moderated t statistics correlated with the comparison ordinary-least-squares t statistics (Spearman rho = 0.97–0.98), every OLS-supported gene had the same direction under limma-voom, and 86 of 131 caudate and 1631 of 2102 putamen OLS-supported genes were also FDR-supported under limma-voom. Adding sequencing run as a fixed effect within region (9–11 additional parameters) reduced the FDR-supported counts to 78 (caudate) and 1126 (putamen) while preserving every direction (*t*-statistic rho = 0.89–0.90; 90–100% of primary genes remained nominally significant), and a random-block duplicateCorrelation model [41] (consensus correlations −0.01 to 0.19) gave 157 and 1667 genes with rho = 0.97–0.99. The variant-specific discovery counts (Supplementary Figure S5B) and statistic correlations (Supplementary Figure S5C) summarize this sensitivity, while the primary-versus-run-adjusted comparisons show the weaker concordance in the NAc (Supplementary Figure S5D). Camera-supported immune sets in the caudate and putamen were retained under both adjustments (18–20 of 20 and 19 of 19 sets). In the NAc, by contrast, run-adjusted statistics correlated poorly with the primary model (rho = 0.31; camera-supported sets 13 to 0), so NAc results are reported as not robust. Nominal *p* values were near their null expectation in the NAc (1060 genes at *p* < 0.05 versus 1040 expected; Storey π_0_ = 0.86 [44]) and progressively enriched in the caudate (2189; π0 = 0.80) and putamen (4812; π_0_ = 0.55) despite similar sample sizes, residual variance, and RNA-quality metrics across regions (Supplementary Figure S5E). Caudate FDR-supported genes were 98% direction-concordant in the putamen, whereas putamen genes were largely discordant in the NAc (31% concordant), indicating that the dorsal gradient is a property of the data rather than of sample size.

### 3.3. Immune-Reference Deconvolution Is Uninformative for Striatal Parenchyma

Because the LM22 leukocyte signature was developed for blood and tumor leukocyte mixtures, we treated CIBERSORT-LM22, CD4 memory-state, and cerebrospinal-fluid single-cell MuSiC analyses as sensitivity analyses rather than as tests of immune-cell abundance (Supplementary Figures S6–S8). Mean IOBR-CIBERSORT LM22 profiles were similar between schizophrenia and control samples in all regions (Supplementary Figure S6A); among 66 region-by-cell-type comparisons, none passed regional FDR < 0.10 (minimum q = 0.85; Supplementary Figure S6B). Resting and activated CD4 memory fractions (Supplementary Figure S7A) and their activation balance (Supplementary Figure S7B) were examined alongside marker and projection scores. The adjusted models produced nominal putamen effects (*p* = 0.025–0.043) with regional q = 0.128 (Supplementary Figure S7C). The marker-gene heatmap showed heterogeneous expression across diagnosis-region groups rather than a uniform CD4-associated shift (Supplementary Figure S7D). The CD4 activation balance correlated with TNFA/NF-kB scores only nominally (Spearman r = −0.27, q = 0.24; Supplementary Figure S7E). The CNS-adjacent CSF reference contained 6684 immune cells across 27 states (Supplementary Figure S8A); its bulk projections were dominated by the broad myeloid/microglial class in both groups (Supplementary Figure S8B). The general model yielded region-level signals for NAc CD4 T-helper (q = 0.081) and putamen plasma-cell proportions (q = 0.066; Supplementary Figure S8C). CD4-conditioned estimates were concentrated in helper, regulatory, and proliferative states (Supplementary Figure S8D), with region-level differences in putamen regulatory and proliferative T-cell balance (q = 0.091; Supplementary Figure S8E). None of these CSF-reference findings survived global FDR correction. Because these references are not matched to brain parenchyma, the null and nominal results do not establish the presence or absence of immune-cell infiltration or exclude leukocyte-composition changes. They leave the cellular source of the Hallmark inflammatory signal unresolved.

### 3.4. A Human Brain Single-Nucleus Reference Indicates Lower Oligodendrocyte-Lineage Weight in Schizophrenia Putamen

We next moved to a tissue-matched human brain single-nucleus reference. HPA/Siletti cluster profiles yielded 70–80 expressed markers for each of seven broad classes: MSN, interneuron, astrocyte, oligodendrocyte, OPC, microglia, and vascular cells (Figure 2A). Non-negative least-squares weights reproduced the expected dominance of neuronal, astrocytic, oligodendroglial, microglial, and vascular profiles in the group-mean profiles (Figure 2B), while sample-level distributions showed variation within diagnostic groups (Figure 2C).

Putamen OPC weight was lower in schizophrenia than control samples (means 0.0155 versus 0.0269; adjusted arcsine-scale beta = −0.049, 95% CI −0.080 to −0.017, *p* = 0.003, regional q = 0.018, global q = 0.053), and putamen oligodendrocyte weight was also lower (beta = −0.030, 95% CI −0.058 to −0.002, *p* = 0.039, regional q = 0.12; Figure 2C,D). Both effects persisted with sequencing run as a fixed effect (OPC beta = −0.037, *p* = 0.037, q = 0.074; oligodendrocyte beta = −0.038, *p* = 0.015, q = 0.045). Because NNLS weights depend on the reference, its scale, and marker definitions, we re-estimated composition with three further approaches (Figure 2E): row-scaled linear-scale NNLS with the same reference, nu-support-vector regression (the IOBR implementation of CIBERSORT) with the same 523-gene signature matrix, and MuSiC with an independent eight-donor human NAc single-nucleus reference (19,892 nuclei; Tran et al. [29]). All three estimated lower putamen oligodendrocyte weight in schizophrenia (beta = −0.047, *p* = 0.004, q = 0.031; beta = −0.043, *p* = 0.023; and beta = −0.037, *p* = 0.054, respectively), and the combined oligodendrocyte-lineage weight (OPC plus oligodendrocyte) was lower under all four estimators (*p* = 0.0004, 0.004, 0.019, and 0.057). Oligodendrocyte estimates agreed across methods (Spearman rho = 0.70–0.95 across 215 samples; 0.86–0.95 among log-scale NNLS, CIBERSORT, and MuSiC), whereas OPC estimates did not (rho = −0.01 to 0.60): OPC weights were zero in most samples under the linear-scale methods and averaged 0.1% with MuSiC (Supplementary Figure S9C). Mean reference weights also differed substantially across estimators, with no vascular class represented in the Tran reference (Supplementary Figure S9D). In simulated mixtures, the log-scale estimator recovered the OPC rank order (r = 0.95) but overestimated its level (slope 1.7; Supplementary Figure S9A), and leave-one-cluster-out perturbation showed that the OPC-specific attribution depended on including the committed-oligodendrocyte-precursor cluster in the OPC class, without which the same effect appeared as an oligodendrocyte reduction (*p* = 0.005; Supplementary Figure S9B). We therefore interpret the putamen signal as a reduction of oligodendrocyte-lineage reference weight whose attribution to precursors versus mature oligodendrocytes is not resolved by bulk deconvolution.

### 3.5. Exploratory Pathway and Directional-Signature Analyses Prioritize Candidate Molecular Axes

The diagnosis-specific Hallmark analysis identified broad systems-level changes. We next tested whether a curated pathway workflow resolved these changes into interpretable neuropsychiatric axes; because these analyses select and re-test gene sets within the same data, they are exploratory. Rank-based scoring of 1669 Elsevier pathways in the pooled psychosis group (schizophrenia and bipolar psychosis combined) identified 30 FDR-supported pathways in the putamen and 13 in the caudate, but none in the NAc (Supplementary Figure S10B). Selected pathways included dopamine metabolism, glutamate–calcium stress, immune-NF-kB signaling, Activin/BMP-SMAD biology, and APP processing (Supplementary Figure S10A). Representative scores showed higher dopamine-metabolism scores in the putamen and presynaptic-glutamate scores in the caudate but lower IL6ST-STAT5B scores in the putamen, in pooled psychosis (Supplementary Figure S10C). The regional volcano plots showed no FDR-supported pathway in the NAc (Supplementary Figure S10D) and both positive and negative pathway effects in the caudate (Supplementary Figure S10E) and putamen (Supplementary Figure S10F). Gene-level analysis of 168 members of 12 selected pathways prioritized INHBA, SMAD1, TH, PSEN1, GRM8, IL6, XBP1, and KAT5 as candidate genes, with pathway membership linking these candidates to the selected molecular axes (Supplementary Figure S11A). Regional coefficients showed increased INHBA and SMAD1 and reduced TH, PSEN1, and KAT5 in the dorsal regions (Supplementary Figure S11B); the evidence plot combined membership count with the minimum gene-level *p* value (Supplementary Figure S11C). The volcano plots showed weaker NAc effects (Supplementary Figure S11D), contrasting with the larger TH and INHBA signals in the caudate (Supplementary Figure S11E) and the prominent INHBA and SMAD1 signals in the putamen (Supplementary Figure S11F).

Direction-aware aggregation reduced these candidate genes to five signatures whose gene membership came from the selected pathways and whose gene-wise directions were assigned from the sign of each gene’s strongest pooled-psychosis coefficient across regions. The directions were therefore data-informed rather than prespecified, and signature *p* values in the same data are exploratory. To assess direction selection conditional on the chosen genes, we repeated direction assignment, scoring, and regional modeling under 1000 donor-level permutations of the psychosis label while holding signature membership fixed. The upstream pathway screening and candidate-gene selection were not repeated; the permutation *p* values therefore do not correct for those selection steps. Selection shifted the null t statistic by +1.1 to +1.4, but the Activin–SMAD, dopamine–adenosine, glutamate–calcium, and APP-processing signatures remained supported in caudate and putamen (selection-aware *p* ≤ 0.010; FDR ≤ 0.013), whereas NAc effects weakened (immune-NF-kB, *p* = 0.017, FDR = 0.085; dopamine–adenosine, *p* = 0.094; Figure 3A and Supplementary Figure S3C). Directions assigned in the caudate reproduced the putamen effects (*p* < 10^−5^) and vice versa (*p* ≤ 0.013), although the regions share donors and this is not an independent replication (Supplementary Figure S3D). Estimating schizophrenia and bipolar psychosis separately showed that the pooled effects were driven by schizophrenia (beta = 0.24–0.52 in caudate and putamen, *p* ≤ 0.009); bipolar-psychosis estimates had the same direction but wider intervals that generally included zero (beta = 0.18–0.46), and the two diagnoses differed significantly only for putamen Activin–SMAD (*p* = 0.019) and dopamine–adenosine (*p* = 0.011) (Figure 3B). The caudate and putamen each contained four signatures at selection-aware q < 0.10, compared with one in the NAc (Figure 3C). The schematic in Figure 3D is an evidence-organizing representation of candidate axes, not a causal network, and the Activin–SMAD and APP axes remain more hypothesis-generating than the dopamine, glutamate, and inflammatory components.

### 3.6. Genetic-Regulatory and Cell-Marker Evidence Separates Risk Support from Transcriptomic State

We finally integrated the signature genes with GTEx v8 brain independent cis-eQTL records and GWAS Catalog psychiatric mapped-gene associations. CACNA1C carried the broadest psychiatric GWAS support (Figure 4A), while GRM8, CACNA2D1, ADAM10, ADAM17, and XBP1 showed varying brain eQTL or combined support (Figure 4B). The axis-level summary distinguished genes with eQTL-only, GWAS-only, combined, or neither evidence layer, with the largest combined-support component in the glutamate–calcium axis (Figure 4C). In contrast, INHBA, TH, SMAD1, PSEN1, and KAT5 were prioritized mainly by the striatal transcriptomic models. The analysis therefore separated genes supported as external regulatory-risk candidates from genes that primarily marked the observed postmortem disease state. It is gene-level prioritization and did not constitute variant-level colocalization.

Curated marker enrichment linked dopamine–adenosine genes to D2 MSN and dopaminergic-axon programs and immune-NF-kB genes to microglial markers (Figure 4D). Pooled-psychosis marker-score modeling identified reduced D1 MSN scores in the caudate and putamen, increased dopaminergic-axon and microglial scores in the putamen, reduced oligodendrocyte scores in the putamen, and increased vascular scores in the caudate (Figure 4E). External HPA/Siletti markers were derived and rescored in the same samples (Supplementary Figure S12A). Their limited overlap with the internal panels showed that the marker definitions were not identical (Supplementary Figure S12B). The external scores reproduced reduced oligodendrocyte and OPC marker scores in the putamen (Supplementary Figure S12C), and internal-versus-external effects were concordant across 21 matched class–region pairs (Spearman rho = 0.67, *p* = 8.9 × 10^−4^; Supplementary Figure S12D). Because disease effects are re-estimated in the same cohort, this agreement is within-dataset triangulation rather than independent replication; it supports, but does not replace, the explicit composition analysis in Figure 2.

## 4. Discussion

This exploratory re-analysis characterizes region-specific striatal transcriptomic and cellular differences in schizophrenia and bipolar psychosis and grades the evidence for each finding by explicit criteria (Table 1). Schizophrenia showed a marked caudate-to-putamen increase in gene-level burden under limma-voom that was robust to sequencing-run adjustment, accompanied by camera-supported immune–inflammatory Hallmark programs in both dorsal regions. The bipolar-psychosis arm was underpowered: with eight donors and only one FDR-supported gene (CCBE1 in caudate), the broader pattern remained inconclusive; its Hallmark and signature estimates were directionally concordant with schizophrenia but imprecise. We therefore describe pooled-psychosis analyses as schizophrenia-driven rather than transdiagnostic, and we do not claim diagnosis-specific molecular organization.

Findings were graded on four criteria: whether the statistic comes from an established framework with false-discovery control; whether the reference or gene set is matched to the tissue; whether an orthogonal method in the same data agrees; and whether independent external data support the direction (Table 1). Under these criteria, the putamen gene-level burden and the dorsal immune–inflammatory programs are tier 1 findings (established framework, robust to run adjustment, concordant between caudate and putamen); the putamen oligodendrocyte-lineage reduction is tier 2 (four estimators and two references agree, and independent histology reports reduced oligodendrocytes in anterior putamen and caudate in schizophrenia [19,49]); the directional signatures are tier 3 (within-dataset and data-informed, exploratory after direction-selection correction conditional on fixed gene membership); and the eQTL/GWAS annotations are gene-level prioritization evidence rather than tests. Composition estimates and Hallmark tests address different questions (abundance versus state) and are not ranked against each other. Where layers conflict, a reference that is not matched to the tissue is treated as uninformative rather than as counter-evidence: the LM22 null result does not contradict the Hallmark inflammatory signal because LM22 cannot resolve parenchymal immune states.

The immune results therefore separate pathway activity from inferred cellular abundance without resolving its cellular source. TNFA/NF-kB, IL6/JAK/STAT3, and related programs were camera-supported in the caudate and putamen, consistent with region- and state-dependent neuroinflammatory abnormalities in schizophrenia [46,47,48]. Broad LM22 fractions, CD4 memory metrics, and CSF-reference states did not show FDR-supported shifts, but these references are not matched to brain parenchyma and cannot exclude infiltration or microglial activation. The inflammatory signal may reflect transcriptional state within resident cells, low-abundance infiltrating cells, or postmortem and medication effects; distinguishing these requires single-nucleus or spatial data with immune resolution.

The composition analysis provides a more tissue-matched cellular result whose direction was stable across four estimators and two references, including an independent multi-donor striatal single-nucleus reference [29]. Its cell-type attribution was not: the log-scale NNLS formulation placed the reduction on OPCs, the linear-scale, CIBERSORT, and MuSiC formulations placed it on mature oligodendrocytes, and OPC estimates disagreed across methods. Because OPCs represent a few percent of striatal nuclei and their markers are among the least expressed in bulk tissue, bulk deconvolution is unlikely to resolve them; we therefore report an oligodendrocyte-lineage reduction. This direction agrees with the internal and external marker-score analyses and with independent histological reports of reduced oligodendrocyte density in the anterior putamen and caudate in schizophrenia [19,49]. NNLS, support-vector, and MuSiC weights remain relative reference contributions rather than histological counts, and a confirmatory test requires striatal single-nucleus or histological data from schizophrenia donors, which were not available to us.

The pathway and signature analyses relate the systems-level findings to candidate molecular axes but are exploratory. Gene membership came from pathways that were themselves selected in this dataset, and gene directions were assigned from the data; the permutation analysis assesses direction-selection effects conditional on fixed signature membership. It does not repeat the preceding pathway screening or gene selection and therefore does not eliminate selection bias from the full workflow. The caudate and putamen signatures remain exploratory within-dataset summaries requiring validation in independent disease samples. Dopamine–adenosine and glutamate–calcium programs are consistent with established striatal models of schizophrenia [50,51,52,53,54,55,56,57,58,59,60], and NF-kB-related effects agree with a broader literature on neuroinflammatory abnormalities [46,47,48]. Activin–SMAD and APP-processing signals recurred across analyses but have weaker direct psychosis evidence; APP-related results should not be interpreted as Alzheimer-type amyloid pathology, and available postmortem and peripheral findings provide only indirect support for altered APP abundance or metabolism in schizophrenia [61,62]. Both axes remain hypotheses for experimental testing.

Genetic-regulatory integration further distinguished trait-associated candidates from state-associated transcripts. CACNA1C and several glutamate–calcium or APP-processing genes had external GWAS or brain eQTL support, whereas INHBA, TH, SMAD1, PSEN1, and KAT5 were nominated mainly by the postmortem expression model. This distinction is prioritization rather than a test of causality: a disease-state transcript need not mediate inherited risk, and a GWAS-mapped gene need not be differentially expressed in adult tissue. Formal colocalization, fine mapping, and allele-aware expression analysis are required before assigning a shared causal variant.

Several limitations define the interpretation. Bulk postmortem RNA-seq cannot separate cell-intrinsic regulation, cell-state change, cellular composition, medication exposure, agonal factors, and technical effects; chronic antipsychotic exposure can itself produce cell-type-specific transcriptional changes in striatal model systems [63]. Sequencing runs were nested within region and, in the NAc, partly aligned with diagnosis, so NAc findings are not robust and the regional gradient is best supported for caudate and putamen; surrogate-variable adjustment (svaseq) removed most signal in these small regional samples and was therefore not used as a primary model. The bipolar-psychosis group was small; direct behavioral, cognitive, and symptom-level phenotypes were unavailable; and all directional-signature results are exploratory. Reference-based composition estimates depend on reference, scale, and marker choice, as shown directly here, and the LM22 and CSF references are not matched to striatal parenchyma. Finally, all findings derive from one cohort and require replication in independent striatal single-nucleus, spatial, or histological datasets.

Together, the analyses place diagnosis-specific gene burden, correlation-aware systems enrichment, brain-resident composition, and genetic annotation on separate evidence tiers. The best-supported chain links schizophrenia-associated caudate and putamen transcriptional remodeling to immune–inflammatory programs and to a putamen oligodendrocyte-lineage reduction. These observations provide testable substrates for future studies linking striatal circuit biology to psychotic symptoms and behavior, while the dopamine–adenosine, glutamate–calcium, Activin–SMAD, and APP-processing axes remain prioritized hypotheses for cross-cohort and experimental validation.

## 5. Conclusions

Schizophrenia-associated transcriptional burden increased from the nucleus accumbens to the caudate and putamen under limma-voom and was robust to sequencing-run adjustment in the dorsal regions. Correlation-aware tests supported immune–inflammatory Hallmark programs in the caudate and putamen, whereas leukocyte-reference deconvolution was uninformative for brain parenchyma. Across four estimators and two single-nucleus references, schizophrenia putamen samples showed a lower oligodendrocyte-lineage reference weight; its attribution to precursors versus mature oligodendrocytes was method-dependent. Bipolar-psychosis results were inconclusive because of sample size, and the directional signatures remain exploratory. The study separates transcriptional state, inferred cellular context, and inherited-risk annotation by explicit evidence tiers and provides prioritized, testable hypotheses for independent striatal single-nucleus and histological validation.

## Figures and Tables

**Figure 1:**
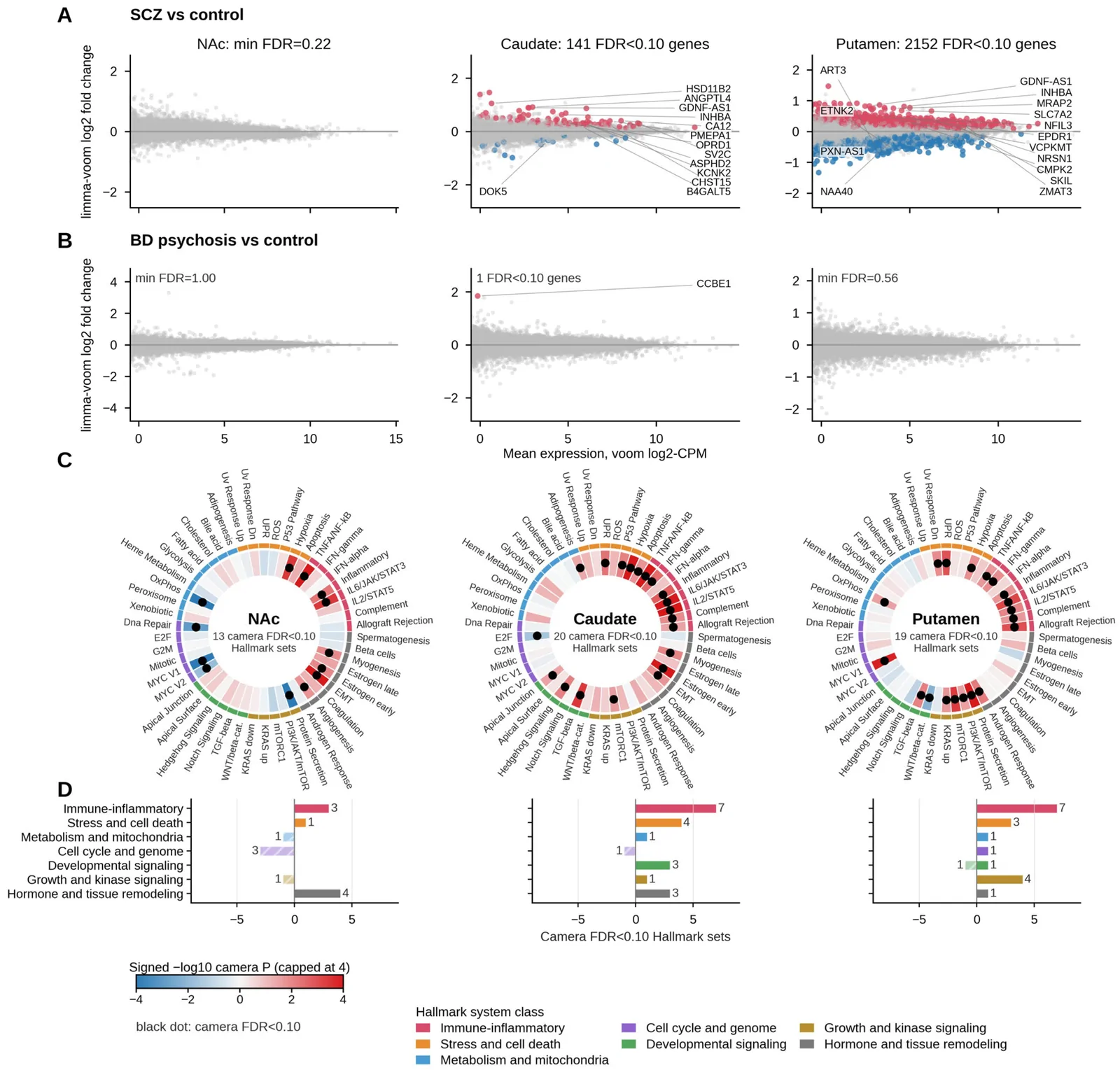
Diagnosis-specific transcriptome (limma-voom) and Hallmark system (camera) discovery. (**A**) Mean expression (voom log_2_-CPM) versus limma-voom log_2_ fold change for schizophrenia versus control in NAc, caudate, and putamen. Red points denote up-regulated and blue points down-regulated genes at FDR < 0.10; grey points denote all other genes. Labels identify the most significant FDR-supported genes, and the panel titles report the number of FDR-supported genes or the minimum FDR in each region. (**B**) Corresponding plots for bipolar disorder with psychosis versus control; panel annotations report the number of FDR-supported genes or the minimum FDR in each region; colors are as in (A). (**C**) Circular heatmaps of schizophrenia Hallmark enrichment across the same 50 gene sets in NAc, caudate, and putamen. Tile color represents the signed −log_10_ camera *p* value (capped at 4; red, enrichment among up-regulated genes; blue, among down-regulated genes), black dots denote camera FDR < 0.10, and the outer ring indicates one of seven systems classes (legend at the bottom). (**D**) Number of camera FDR-supported Hallmark sets by systems class and region; bars to the right count up-regulated sets and hatched bars to the left count down-regulated sets; bar colors follow the systems-class legend. Gene-level coefficients were estimated by region-specific limma-voom models containing simultaneous schizophrenia and bipolar-psychosis indicators and covariates; Hallmark *p* values were obtained from camera with an inter-gene correlation of 0.01 and corrected by the Benjamini–Hochberg procedure within diagnosis-region families.

**Figure 2:**
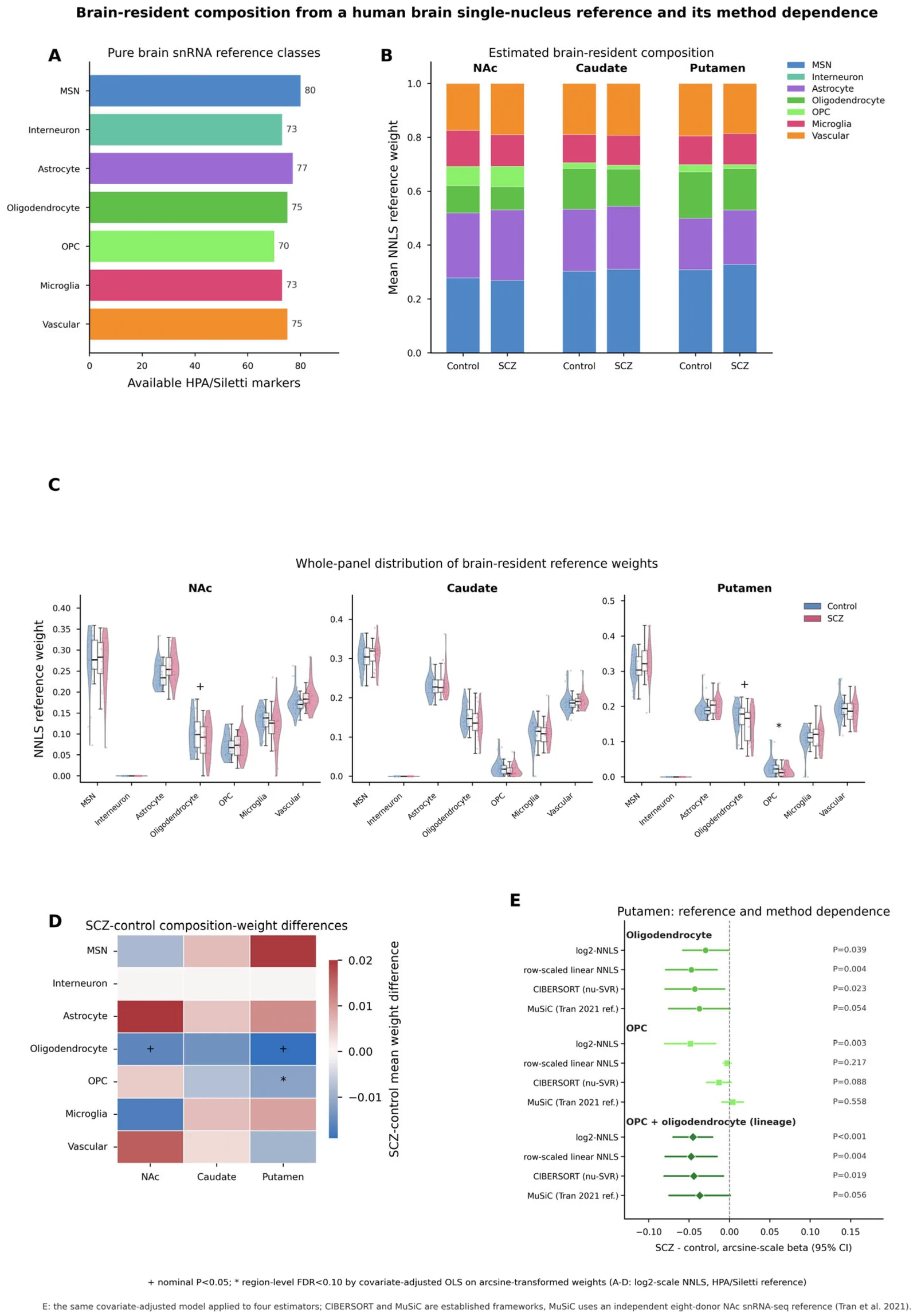
Brain-resident composition estimated from a human brain single-nucleus reference and its dependence on estimator and reference. (**A**) Number of HPA/Siletti marker genes available in GSE202537 for seven broad brain-resident classes. (**B**) Mean normalized NNLS reference weights by diagnosis and region. (**C**) Violin plots with nested boxplots and jittered sample-level weights for all classes. Boxes show median and interquartile range. (**D**) Heatmap of unadjusted schizophrenia-minus-control mean weight differences. (**E**) Putamen schizophrenia-minus-control effects (arcsine-scale beta with 95% confidence intervals) for oligodendrocyte, OPC, and combined oligodendrocyte-lineage weight under four estimators: log_2_-scale NNLS (panels (**A**–**D**)), row-scaled linear-scale NNLS, nu-support-vector regression (IOBR CIBERSORT) with the same HPA/Siletti signature matrix, and MuSiC with an independent eight-donor human NAc single-nucleus reference [29]. Covariate-adjusted tests used OLS models of arcsine-square-root-transformed weights. Plus signs denote nominal *p* < 0.05; asterisks denote region-level FDR < 0.10.

**Figure 3:**
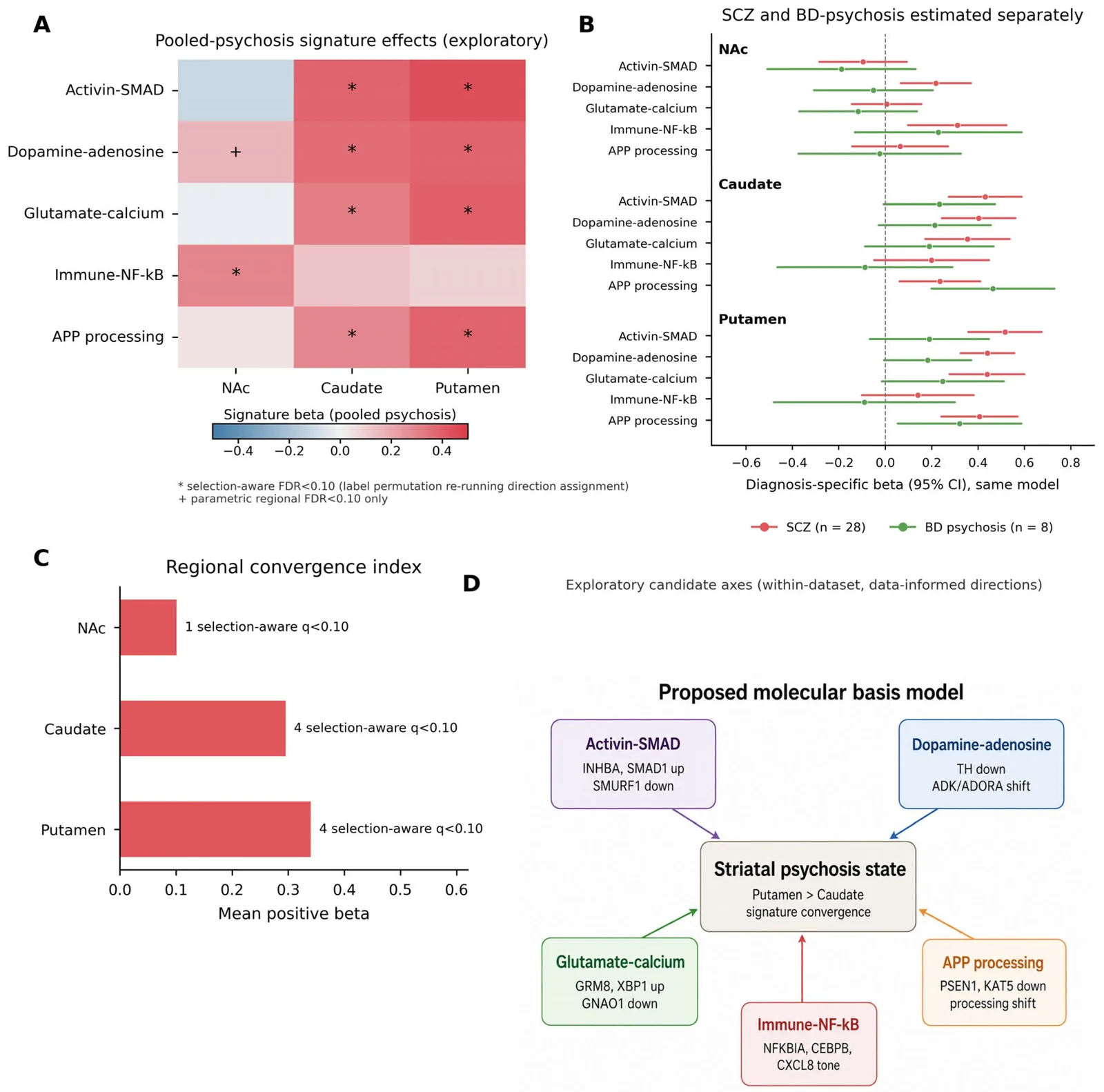
Exploratory directional molecular signatures. (**A**) Heatmap of pooled-psychosis directional-signature coefficients across NAc, caudate, and putamen. Asterisks denote selection-aware FDR < 0.10, obtained by re-running direction assignment, scoring, and regional modeling under 1000 donor-level permutations of the psychosis label with gene membership held fixed; plus signs denote parametric regional FDR < 0.10 only. The permutation addresses direction selection, not the preceding pathway or gene selection. (**B**) Schizophrenia and bipolar-psychosis coefficients (with 95% confidence intervals) estimated separately in the same covariate-adjusted model. (**C**) Regional convergence index, defined as the mean positive signature coefficient; labels report the number of signatures meeting selection-aware q < 0.10. (**D**) Schematic of the five candidate molecular axes. Scores were calculated by z-standardizing component-gene expression, multiplying each gene by its data-informed direction, and averaging within signature. Higher scores indicate stronger agreement with the directional definition and do not imply uniform regulation of every component gene; the axes are within-dataset candidates, not validated mechanisms.

**Figure 4:**
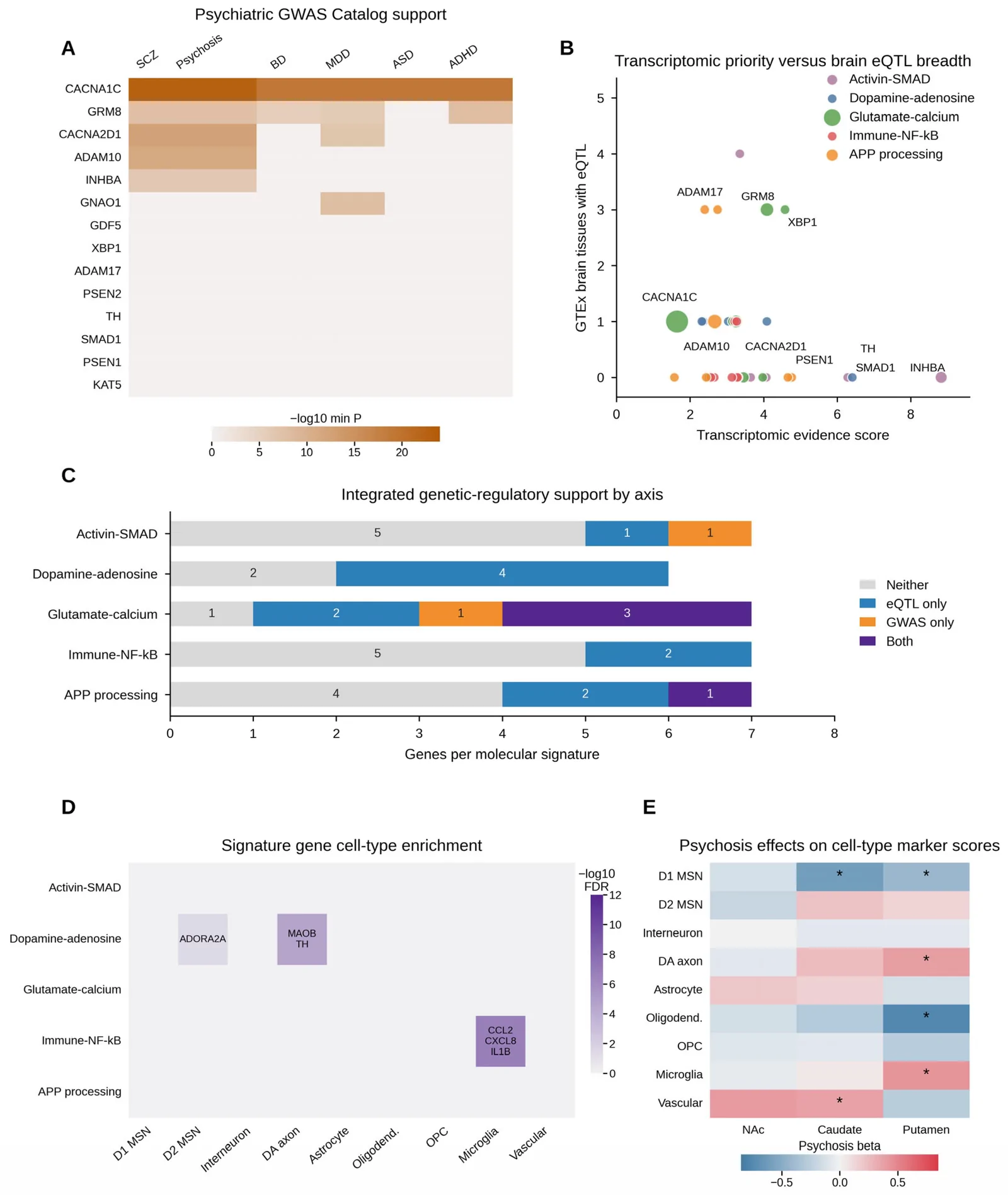
Genetic-regulatory annotation and cell-marker analyses of molecular-signature genes. (**A**) GWAS Catalog mapped-gene support across six psychiatric trait categories, displayed as −log_10_ minimum mapped-gene *p* value. (**B**) Transcriptomic priority score plotted against the number of GTEx v8 brain tissues with independent cis-eQTL support; point size and color encode the figure-defined evidence and molecular-axis annotations. (**C**) Counts of signature genes classified as neither, eQTL only, GWAS only, or both evidence layers. (**D**) One-sided Fisher exact enrichment of signature genes in curated striatal and brain cell-marker panels, displayed as −log_10_ FDR. (**E**) Covariate-adjusted pooled-psychosis marker-score coefficients across NAc, caudate, and putamen. GTEx tissues were caudate, putamen, nucleus accumbens, cortex, and frontal cortex. Gene-level GWAS/eQTL support was not interpreted as variant-level colocalization. Asterisks denote FDR < 0.10.

**Table 1:** Evidence-grading criteria and the tier assigned to each principal finding.

Finding	Established Framework with FDR Control	Tissue-Matched Reference or Gene Set	Orthogonal Agreement Within GSE202537	Independent External Support	Tier
Schizophrenia gene-level burden, putamen > caudate > NAc	Yes (limma-voom; robust to run adjustment in caudate and putamen)	Not applicable	OLS model and voom concordant; caudate-putamen direction concordance 93–98%	Regional burden not previously reported at this resolution	1
Immune–inflammatory Hallmark programs, caudate and putamen	Yes (camera, default correlation; weaker with estimated correlation)	Hallmark sets are tissue-agnostic	Retained under run-adjusted models	Neuroinflammatory literature [46,47,48]	1 (qualified)
Lower oligodendrocyte-lineage weight, schizophrenia putamen	Yes (covariate-adjusted arcsine models, regional FDR)	Yes (human brain single-nucleus references)	Four estimators and two references concordant; marker scores concordant	Histology in anterior putamen and caudate [19,49]	2
OPC-specific attribution of the putamen reduction	Yes	Yes	No (attribution changes with scale and reference)	None available	3
Directional signatures (dopamine–adenosine, glutamate–calcium, Activin–SMAD, APP processing)	Direction-selection permutation conditional on fixed gene membership; upstream selection not corrected	Not applicable	Cross-region transfer within the same donors	Literature for dopamine and glutamate axes [50,51,52,53,54,55,56,57,58,59,60]	3 (exploratory)
NAc Hallmark and signature effects	Yes, but not robust to run adjustment	Not applicable	No	None	Not supported
eQTL/GWAS annotation of signature genes	Not a test	Brain eQTL tissues	Not applicable	GTEx and GWAS Catalog	Annotation only

## Data Availability

The primary striatal transcriptomic data are available from GEO under accession GSE202537. The CSF single-cell reference is available from CELLxGENE Discover (dataset version 0d240f09-c7d4-4420-89ca-376d19663777; collection 573e2e06-8af0-4d96-bfdd-7d64a4bb9c21) and is associated with GEO GSE286518 [37]. The independent NAc single-nucleus reference (SCE_NAc-n8) is distributed by the Lieber Institute for Brain Development [29]. Processed metadata, reference definitions, statistical results, and figure-supporting values for Figure 1, Figure 2, Figure 3, Figure 4 and Supplementary Figures S1–S12, including the limma-voom, camera, run-sensitivity, selection-aware permutation, and alternative-deconvolution result tables, are publicly available as Supplementary Data S1 in Zenodo version 3.0.1. The complete region-specific gene-level model coefficients, *p* values, and false-discovery-rate values supporting Figure 1A,B are available as Supplementary Data S2 in the same record (https://doi.org/10.5281/zenodo.22850201, version 3.0.1; concept DOI https://doi.org/10.5281/zenodo.20741182) [64]. A numbering table in the same record maps the supplementary figure identifiers used by the analysis code to those in this article. The analysis code (Python 3.9 and 3.11 and R 4.5.2) is available at https://github.com/yilin1107/GSE202537-striatal-psychosis/tree/v3.0.1 (accessed on 23 September 2026) and is archived in the same Zenodo record, together with model-verification results and gene-level uncertainty estimates. During the preparation of this manuscript, the authors used OpenAI Codex and Anthropic Claude (accessed on 23 September 2026) to assist with code development, computational workflow implementation, figure preparation, and the drafting, language editing, and formatting of the manuscript text. All tool-assisted code, analytical outputs, and text were reviewed and validated by the authors, who take full responsibility for the content of this publication.

## Supplementary Materials

The following supporting information can be downloaded at: https://www.mdpi.com/article/10.3390/biology15191714/s1, Figure S1, clinical and sampling structure; Figure S2, transcriptome quality control and principal-component structure; Figure S3, schizophrenia versus bipolar-psychosis effects, bipolar-arm power, and selection-aware signature inference; Figure S4, camera and fry versus the permutation test; Figure S5, limma-voom versus OLS, sequencing-run sensitivity, and regional gradient diagnostics; Figure S6, IOBR-CIBERSORT LM22 immune deconvolution; Figure S7, CD4 memory T-cell state analyses; Figure S8, CSF single-cell MuSiC sensitivity analysis; Figure S9, reference, scale, and method dependence of composition estimates; Figure S10, Elsevier pathway analysis; Figure S11, candidate-gene modeling; Figure S12, external HPA/Siletti marker cross-reference; Supplementary Figure Numbering Crosswalk, manuscript-to-archive figure identifiers.

## Abbreviations

The following abbreviations are used in this manuscript:

Abbreviation	Definition
ADHD	attention-deficit/hyperactivity disorder
APP	amyloid precursor protein
ASD	autism spectrum disorder
BD	bipolar disorder
BH	Benjamini–Hochberg
CI	confidence interval
CIBERSORT	cell-type identification by estimating relative subsets of RNA transcripts
CNS	central nervous system
CPM	counts per million
CSF	cerebrospinal fluid
eQTL	expression quantitative trait locus
FDR	false discovery rate
GEO	Gene Expression Omnibus
GTEx	Genotype-Tissue Expression project
GWAS	genome-wide association study
HPA	Human Protein Atlas
IOBR	Immuno-Oncology Biological Research
LM22	leukocyte signature matrix containing 22 immune-cell states
MSigDB	Molecular Signatures Database
MSN	medium spiny neuron
MuSiC	multi-subject single-cell deconvolution
NAc	nucleus accumbens
NF-kB	nuclear factor kappa B
NNLS	non-negative least squares
OLS	ordinary least squares
OPC	oligodendrocyte precursor cell
PCA	principal-component analysis
PMI	postmortem interval
QC	quality control
RIN	RNA integrity number
SCZ	schizophrenia
SMAD	suppressor of mothers against decapentaplegic
snRNA	single-nucleus RNA
TMM	trimmed mean of M values
TOD	time of death
