# Supplementary material for: Systems-Level Transcriptomic and Cellular Architecture of the Human Striatum in Schizophrenia and Bipolar Psychosis
Source: Biology (Basel). 2026 Sep 26;15(19):1714. doi: 10.3390/biology15191714 (PMC13663597; doi:10.3390/biology15191714)
Supplement: Supplementary file 1 [file biology-15-01714-s001.zip › Figure-S11.pdf]

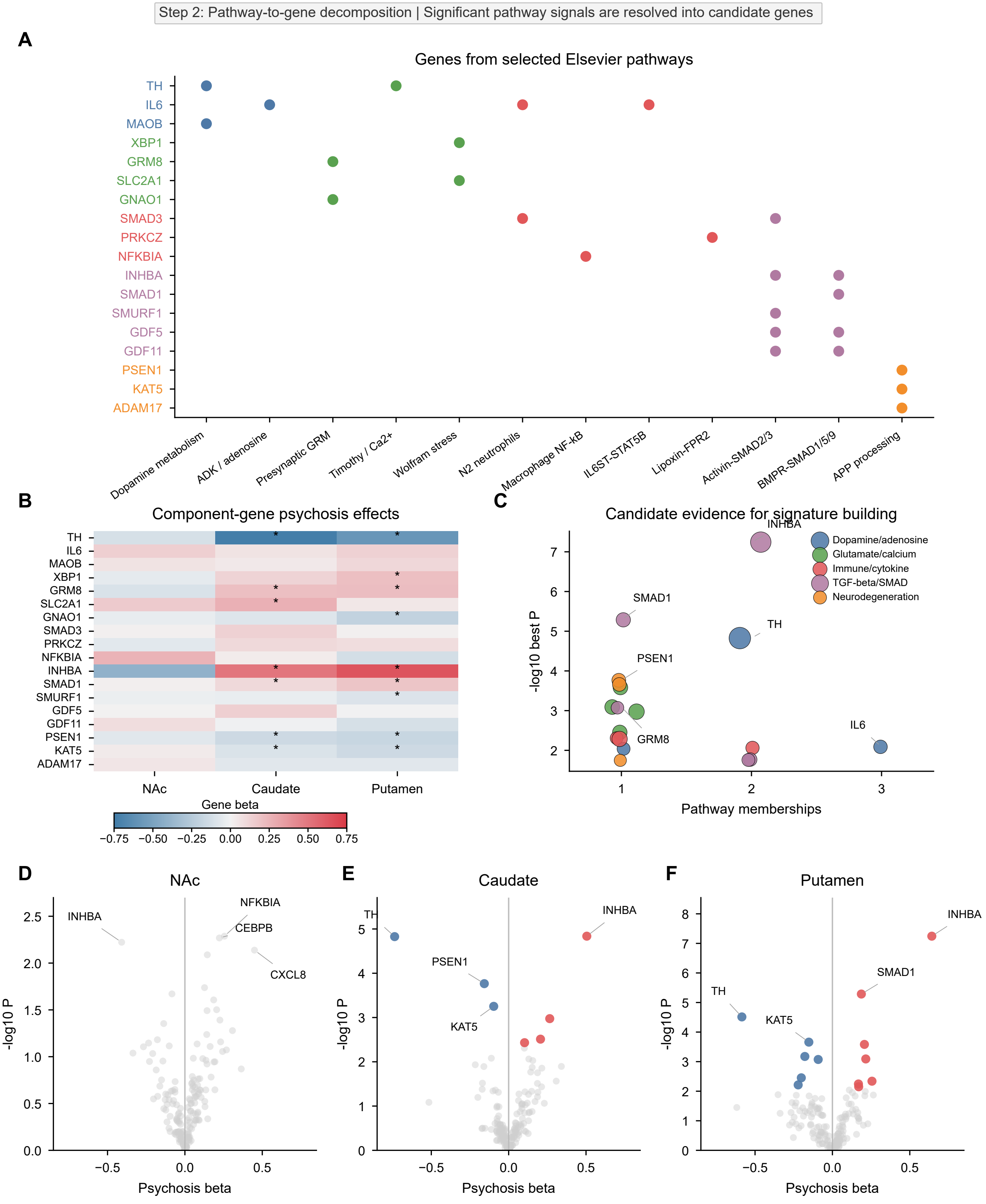

Step 2: Pathway-to-gene decomposition | Significant pathway signals are resolved into candidate genes
A
Genes from selected Elsevier pathways
TH
IL6
MAOB
XBP1
GRM8
SLC2A1
GNAO1
SMAD3
PRKCZ
NFKBIA
INHBA
SMAD1
SMURF1
GDF5
GDF11
PSEN1
KAT5
ADAM17
Dopamine metabolism
ADK / adenosine
Presynaptic GRM
Timothy / Ca2+
Wolfram stress
N2 neutrophils
Macrophage NF-kB
IL6ST-STAT5B
Lipoxin-FPR2
Activin-SMAD2/3
BMPR-SMAD1/5/9
APP processing
B
Component-gene psychosis effects
NAc
Caudate
Putamen
Gene beta
−0.75 −0.50 −0.25 0.00 0.25 0.50 0.75
C
Candidate evidence for signature building
Dopamine/adenosine
Glutamate/calcium
Immune/cytokine
TGF-beta/SMAD
Neurodegeneration
-log10 best P
Pathway memberships
INHBA
SMAD1
TH
PSEN1
GRM8
IL6
D
NAc
E
Caudate
F
Putamen
-log10 P
Psychosis beta
INHBA
NFKBIA
CEBPB
CXCL8
TH
PSEN1
KAT5
SMAD1
