# Supplementary material for: Systems-Level Transcriptomic and Cellular Architecture of the Human Striatum in Schizophrenia and Bipolar Psychosis
Source: Biology (Basel). 2026 Sep 26;15(19):1714. doi: 10.3390/biology15191714 (PMC13663597; doi:10.3390/biology15191714)

Bipolar psychosis versus schizophrenia effects, BD-arm power, and selection-aware signature inference

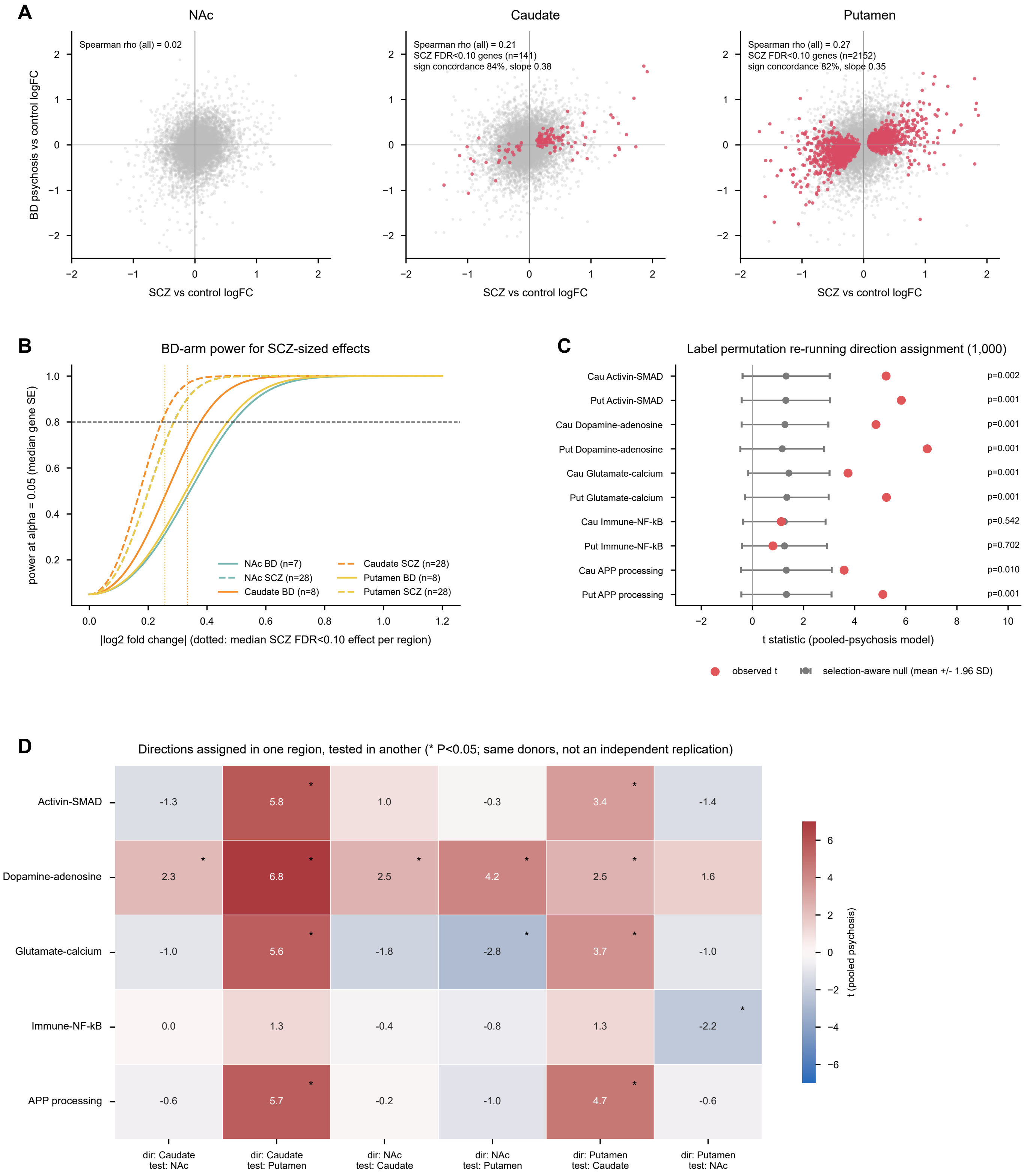

Supplement: Supplementary file 1 [file biology-15-01714-s001.zip › Figure-S3.pdf]
