# Supplementary material for: Systems-Level Transcriptomic and Cellular Architecture of the Human Striatum in Schizophrenia and Bipolar Psychosis
Source: Biology (Basel). 2026 Sep 26;15(19):1714. doi: 10.3390/biology15191714 (PMC13663597; doi:10.3390/biology15191714)

Correlation-aware Hallmark gene-set tests (camera, fry) compared with the gene-sampling permutation test

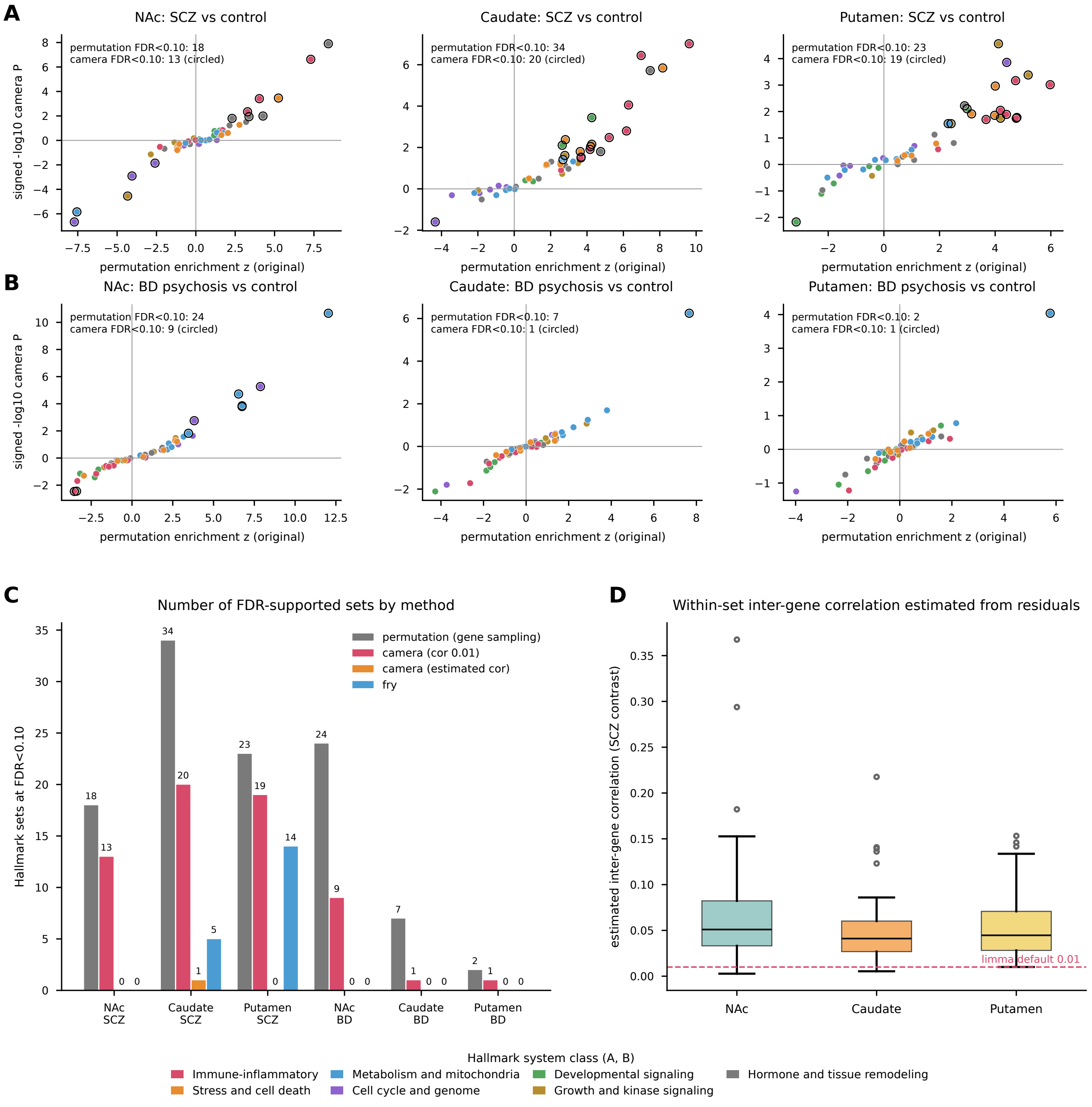

Supplement: Supplementary file 1 [file biology-15-01714-s001.zip › Figure-S4.pdf]
