# Supplementary material for: Systems-Level Transcriptomic and Cellular Architecture of the Human Striatum in Schizophrenia and Bipolar Psychosis
Source: Biology (Basel). 2026 Sep 26;15(19):1714. doi: 10.3390/biology15191714 (PMC13663597; doi:10.3390/biology15191714)
Supplement: Supplementary file 1 [file biology-15-01714-s001.zip › Figure-S5.pdf]

Robustness of the gene-level results: limma-voom versus OLS, sequencing-run adjustment, regional gradient

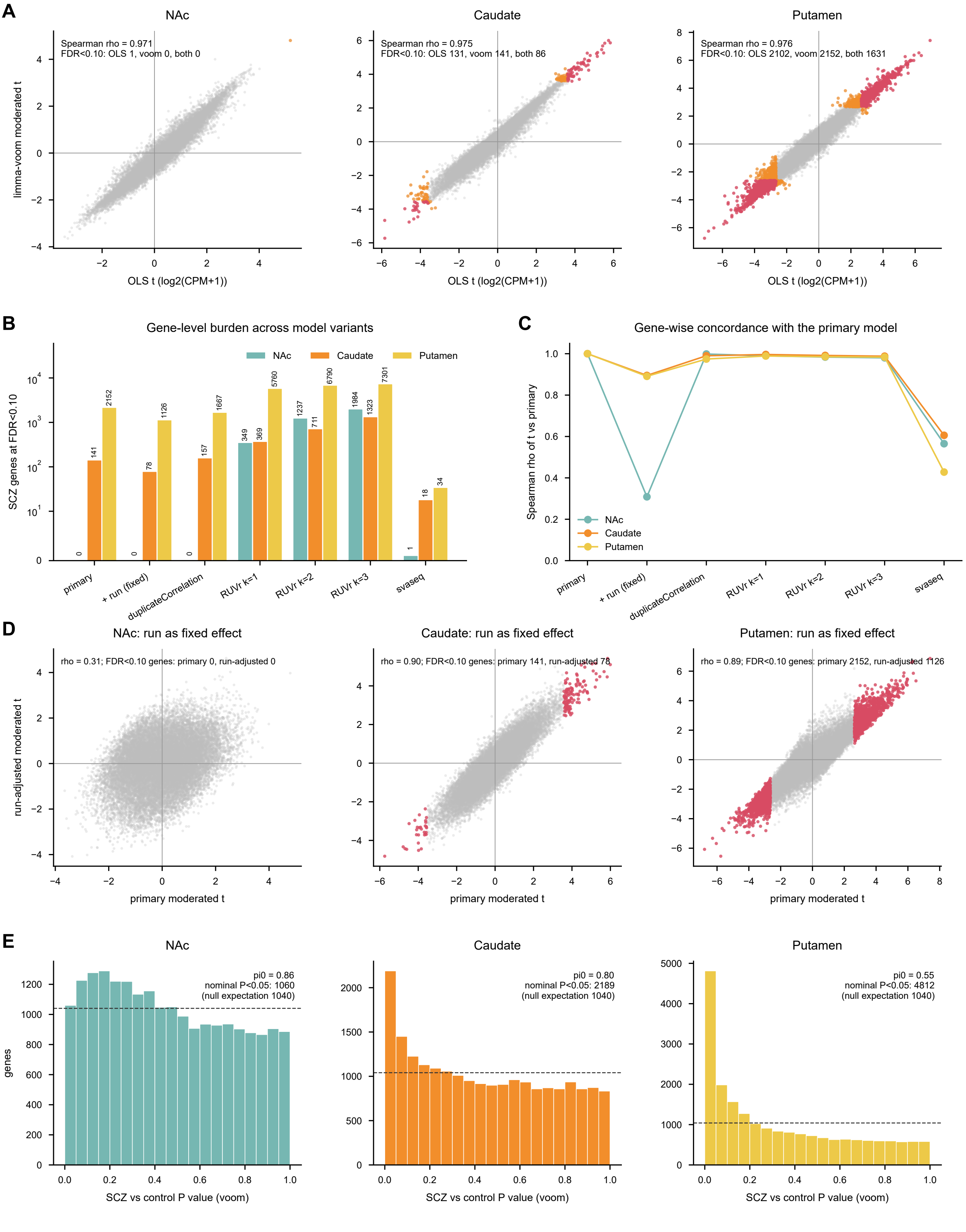

Cross-region concordance of FDR<0.10 genes (sign): caudate->putamen 98%, putamen->caudate 93%, putamen->NAc 31%, caudate->NAc 43%
