# Supplementary material for: Systems-Level Transcriptomic and Cellular Architecture of the Human Striatum in Schizophrenia and Bipolar Psychosis
Source: Biology (Basel). 2026 Sep 26;15(19):1714. doi: 10.3390/biology15191714 (PMC13663597; doi:10.3390/biology15191714)
Supplement: Supplementary file 1 [file biology-15-01714-s001.zip › Figure-S9.pdf]

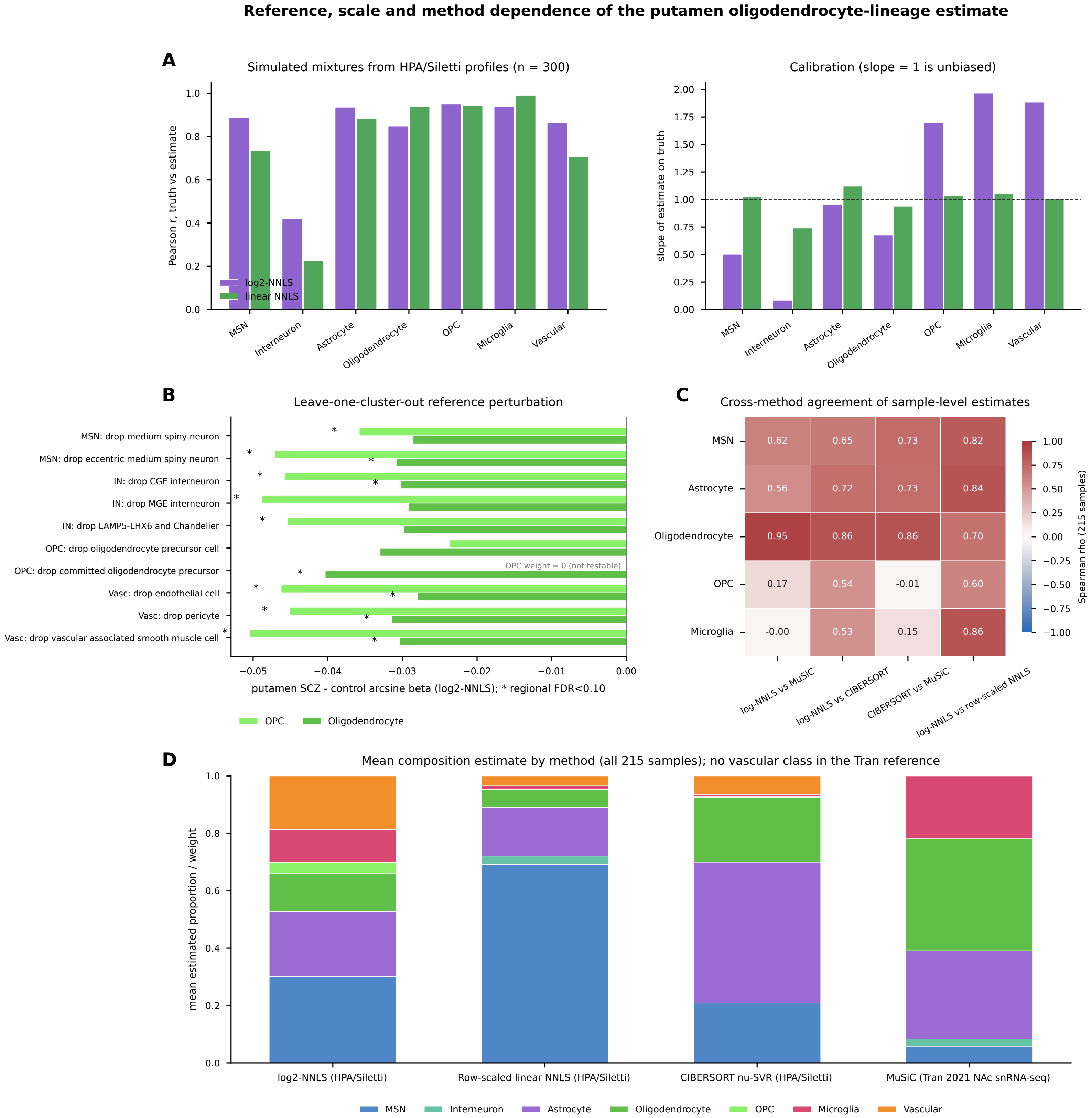
Reference, scale and method dependence of the putamen oligodendrocyte-lineage estimate
A
Simulated mixtures from HPA/Siletti profiles (n = 300)
Pearson r, truth vs estimate
log2-NNLS
linear NNLS
MSN
Interneuron
Astrocyte
Oligodendrocyte
OPC
Microglia
Vascular
Calibration (slope = 1 is unbiased)
slope of estimate on truth
B
Leave-one-cluster-out reference perturbation
MSN: drop medium spiny neuron
MSN: drop eccentric medium spiny neuron
IN: drop CGE interneuron
IN: drop MGE interneuron
IN: drop LAMP5-LHX6 and Chandelier
OPC: drop oligodendrocyte precursor cell
OPC: drop committed oligodendrocyte precursor
Vasc: drop endothelial cell
Vasc: drop pericyte
Vasc: drop vascular associated smooth muscle cell
OPC weight = 0 (not testable)
putamen SCZ - control arcsine beta (log2-NNLS); * regional FDR<0.10
OPC
Oligodendrocyte
C
Cross-method agreement of sample-level estimates
MSN 0.62 0.65 0.73 0.82
Astrocyte 0.56 0.72 0.73 0.84
Oligodendrocyte 0.95 0.86 0.86 0.70
OPC 0.17 0.54 -0.01 0.60
Microglia -0.00 0.53 0.15 0.86
log-NNLS vs MuSiC
log-NNLS vs CIBERSORT
CIBERSORT vs MuSiC
log-NNLS vs row-scaled NNLS
Spearman rho (215 samples)
D
Mean composition estimate by method (all 215 samples); no vascular class in the Tran reference
mean estimated proportion / weight
log2-NNLS (HPA/Siletti)
Row-scaled linear NNLS (HPA/Siletti)
CIBERSORT nu-SVR (HPA/Siletti)
MuSiC (Tran 2021 NAc snRNA-seq)
MSN
Interneuron
Astrocyte
Oligodendrocyte
OPC
Microglia
Vascular
