# Supplementary material for: Systems-Level Transcriptomic and Cellular Architecture of the Human Striatum in Schizophrenia and Bipolar Psychosis
Source: Biology (Basel). 2026 Sep 26;15(19):1714. doi: 10.3390/biology15191714 (PMC13663597; doi:10.3390/biology15191714)
Supplement: Supplementary file 1 [file biology-15-01714-s001.zip › Supplementary Figures.pdf]

Supplementary Figures

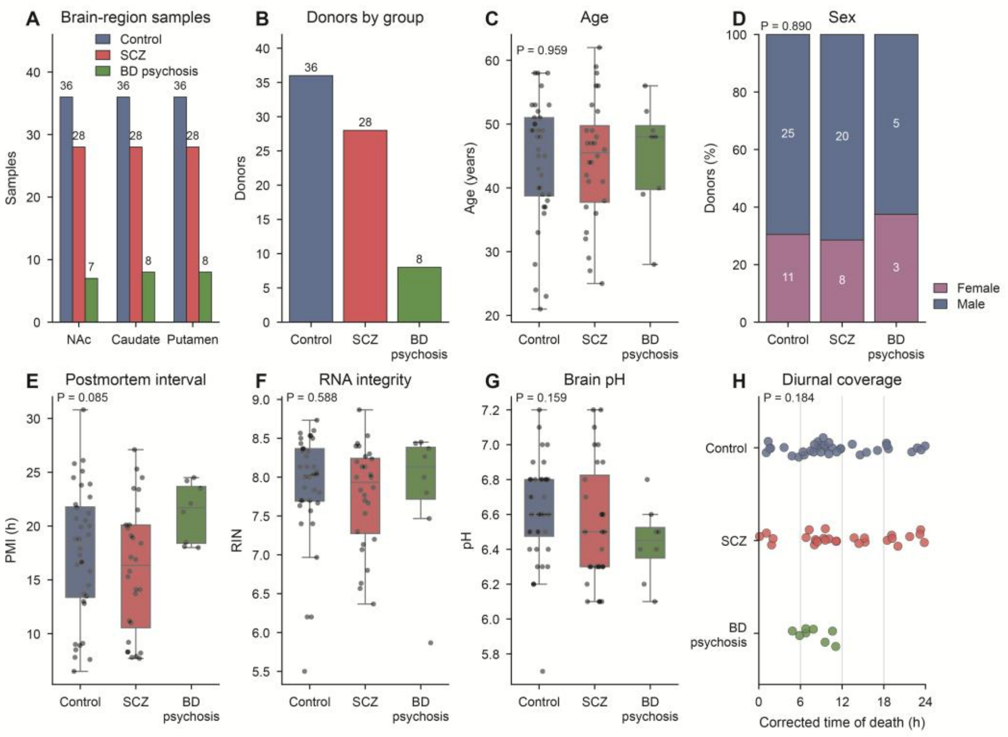

Figure S1. Clinical and sampling structure of the GSE202537 cohort. (A) RNA-seq sample counts by region and diagnostic group. (B) Donor counts by diagnostic group. (C) Age distributions. (D) Sex composition. (E) Postmortem interval. (F) RNA integrity number. (G) Brain pH. (H) Corrected time of death on a 24-hour scale. Boxplots show median and interquartile range with individual donors. Continuous variables were tested by Kruskal-Wallis tests and categorical variables by chi-square tests.

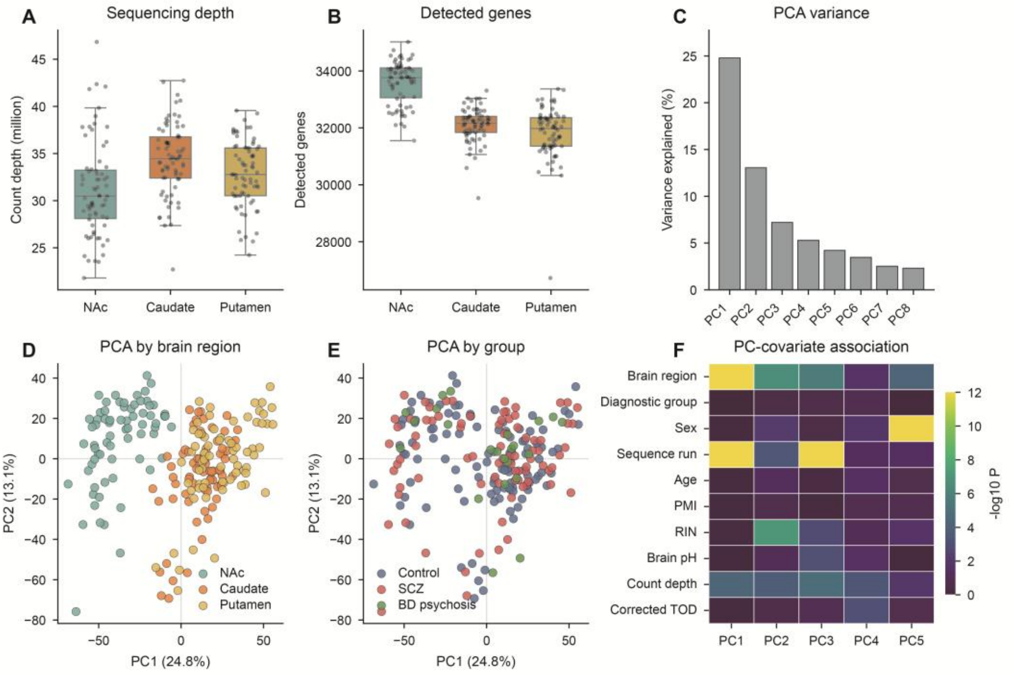

Figure S2. Expression quality control and principal-component structure. (A) Sequencing depth by region. (B) Detected genes by region. (C) Variance explained by principal components 1-8. (D) PCA colored by brain region. (E) PCA colored by diagnostic group. (F) Principal-component associations with biological and technical covariates, displayed as $-\log_{10}$ P values. Continuous covariates were tested by Spearman correlation and categorical covariates by Kruskal-Wallis tests.

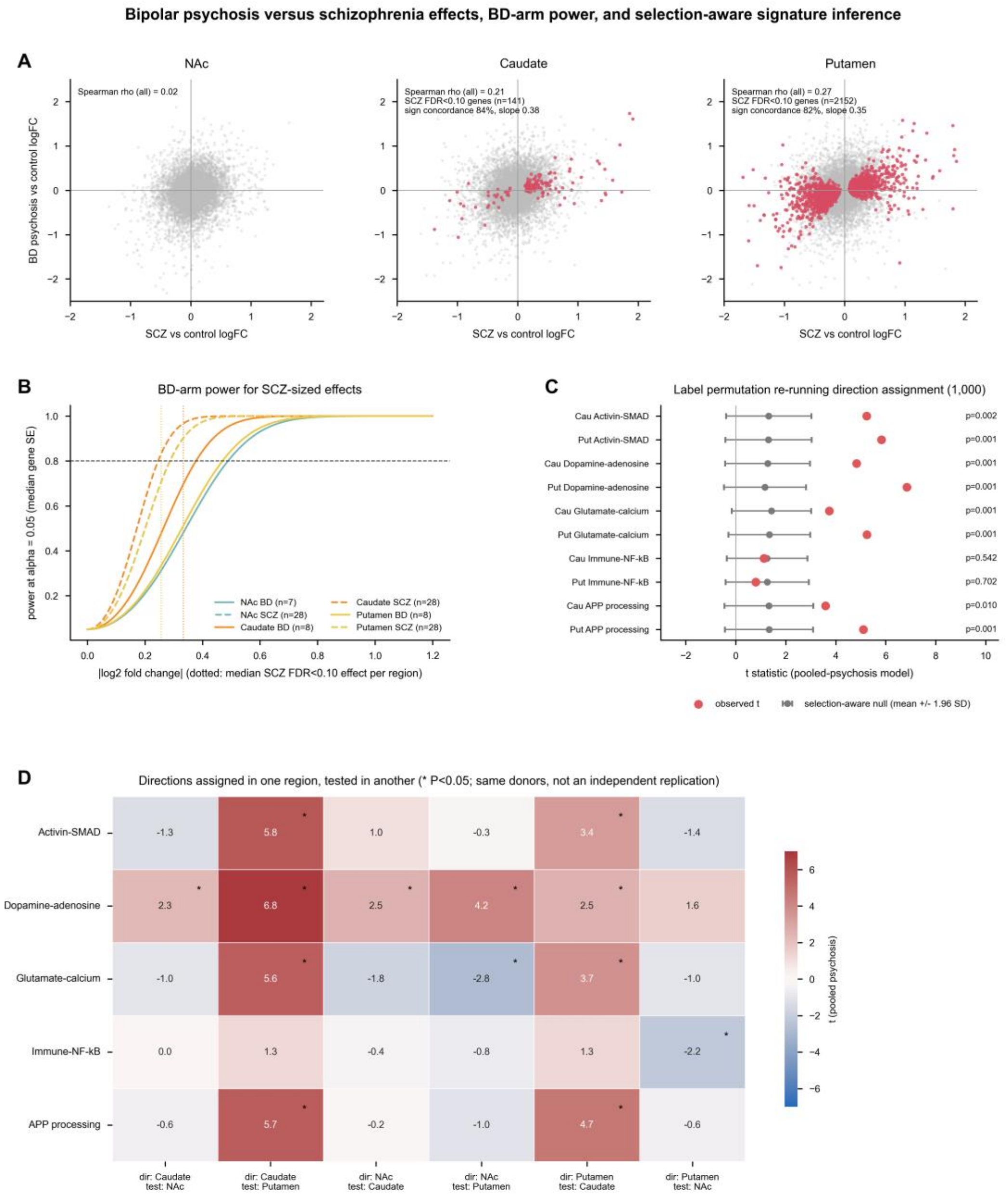

Figure S3. Schizophrenia versus bipolar-psychosis effects, bipolar-arm power, and selection-aware signature inference. (A) Bipolar-psychosis versus schizophrenia log_2 fold changes (limma-voom) for all genes in each region; red, schizophrenia genes at FDR < 0.10, with their sign concordance and regression slope. The estimates and FDR-defined gene sets are identical to those in Figure 1. (B) Power to detect an effect of a given absolute log_2 fold change at alpha = 0.05 in the bipolar-psychosis (solid) and schizophrenia (dashed) arms, using the median gene-wise moderated standard error and degrees of freedom from the same primary R/limma-voom models; dotted vertical lines mark the median absolute effect of schizophrenia FDR-supported genes. (C) Observed pooled-psychosis t statistics for the five signatures in caudate and putamen compared with the null distribution obtained by re-running direction assignment, scoring, and regional modeling under 1,000 donor-level permutations of the psychosis label with signature gene membership held fixed (mean ± 1.96 SD; direction-selection-aware P values at right). The earlier pathway screening and candidate-gene selection were not repeated. (D) Cross-region transfer: t statistics of signature scores whose gene directions were assigned in one region and tested in another;

asterisks denote P < 0.05. Regions share donors, so this is not an independent replication.

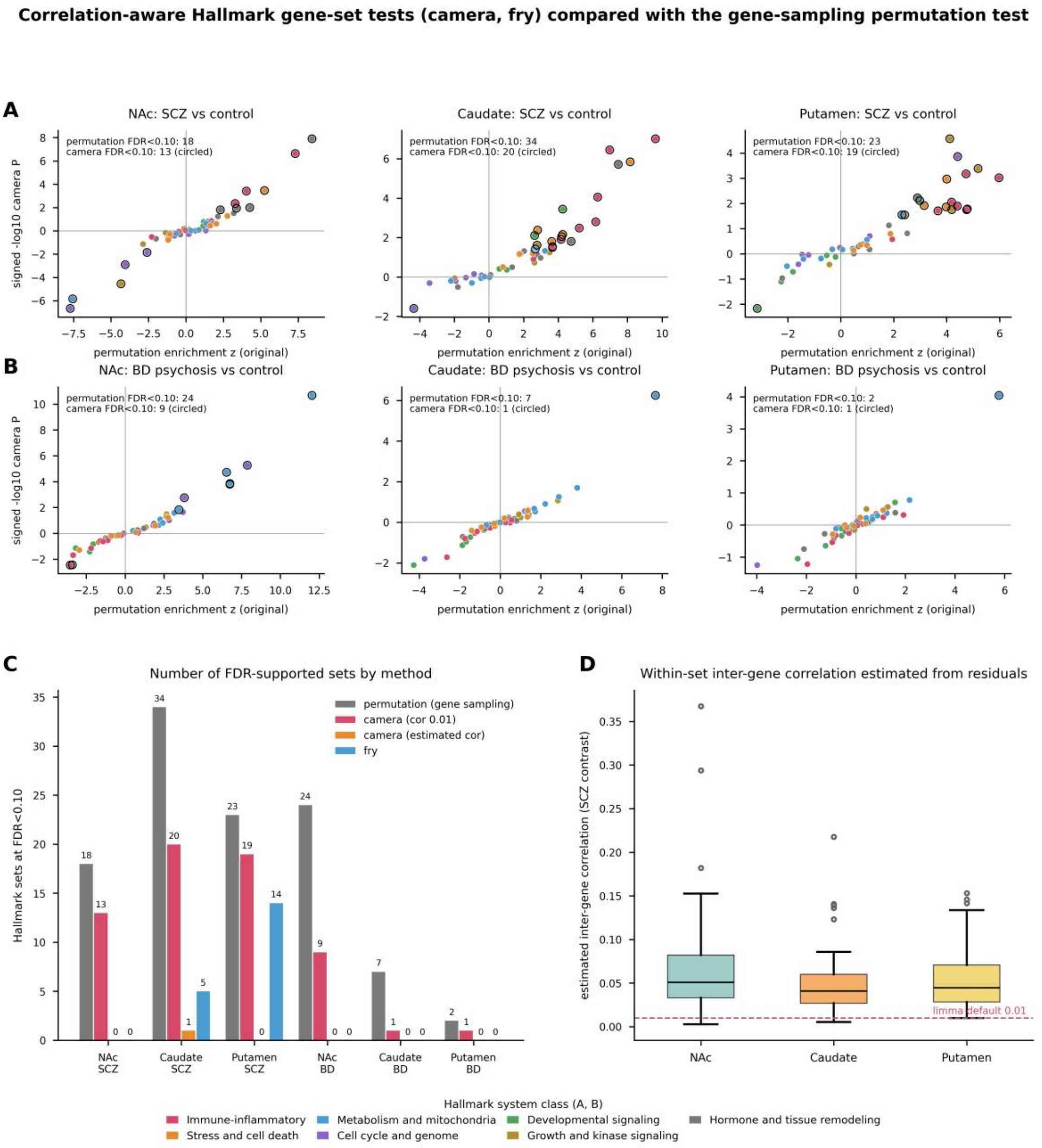

Figure S4. Correlation-aware Hallmark tests compared with the gene-sampling permutation test. (A) Signed -$\log_{10}$ camera P value (inter-gene correlation 0.01) versus the original permutation enrichment z score for each of the 50 Hallmark sets in the schizophrenia contrasts. (B) Corresponding comparisons for bipolar-psychosis contrasts. In both panels, points are colored by systems class and circled when camera FDR < 0.10. (C) Number of FDR-supported sets by method: permutation, camera with correlation 0.01, camera with correlation estimated from residuals, and fry. (D) Distribution of the estimated within-set inter-gene correlation for the schizophrenia contrast; the dashed line marks the default value of 0.01.

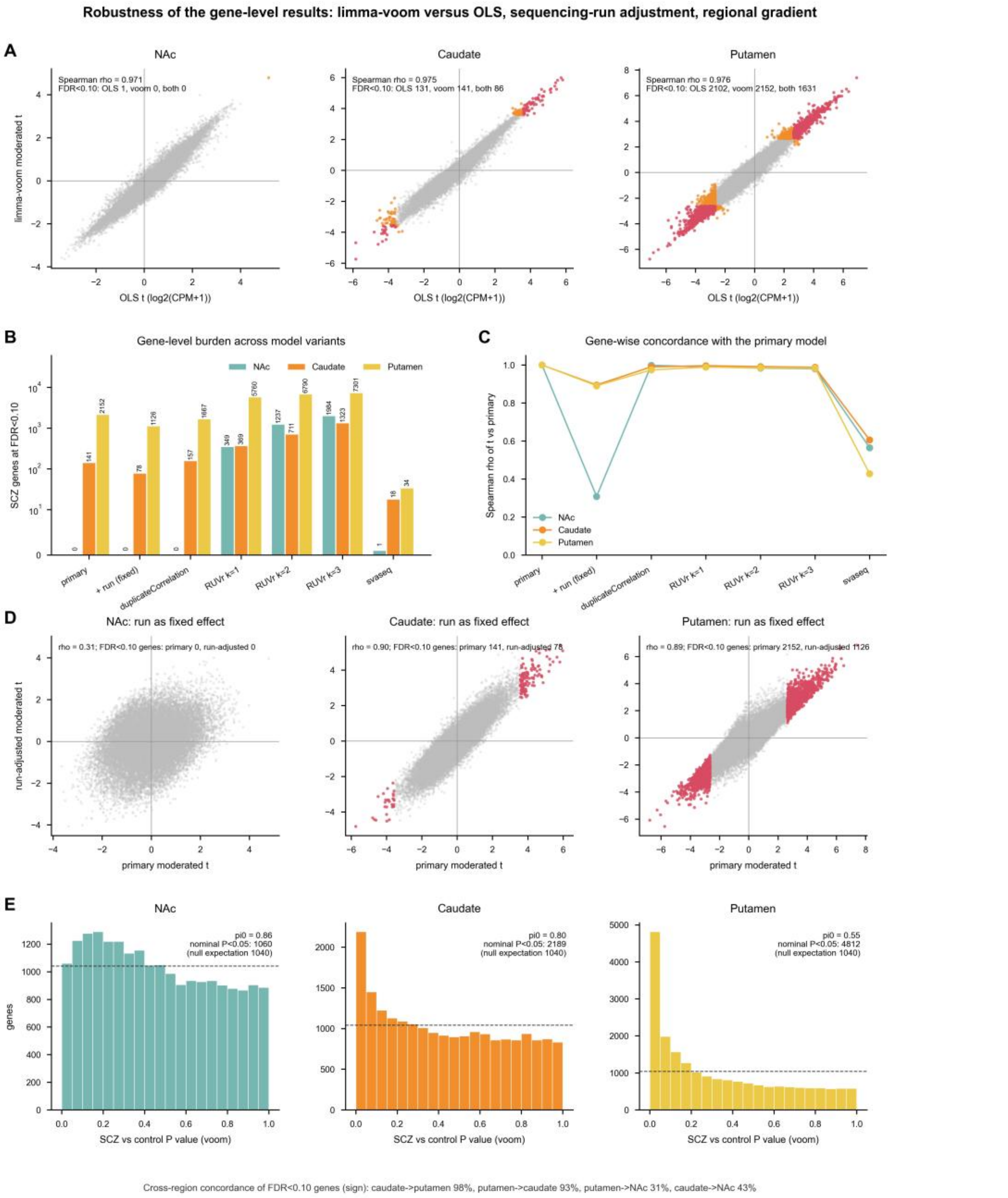

Figure S5. Robustness of the gene-level results. (A) limma-voom moderated t statistics versus the original OLS t statistics for schizophrenia versus control in each region; red, FDR < 0.10 under both models; orange, under one model. (B) Number of schizophrenia genes at FDR < 0.10 under the primary voom model and under sequencing-run adjustments (run as a fixed effect; duplicateCorrelation with run as a random block; RUVr with k = 1-3; svaseq). (C) Spearman correlation of gene-wise t statistics between each variant and the primary model. (D) Primary versus run-adjusted (fixed-effect) t statistics; red, genes at FDR < 0.10 in the primary model. (E) Histograms of schizophrenia P values with Storey's π_0 and the number of nominally significant genes; dashed line, uniform expectation. Cross-region sign concordance of FDR-supported genes is reported below the panels.

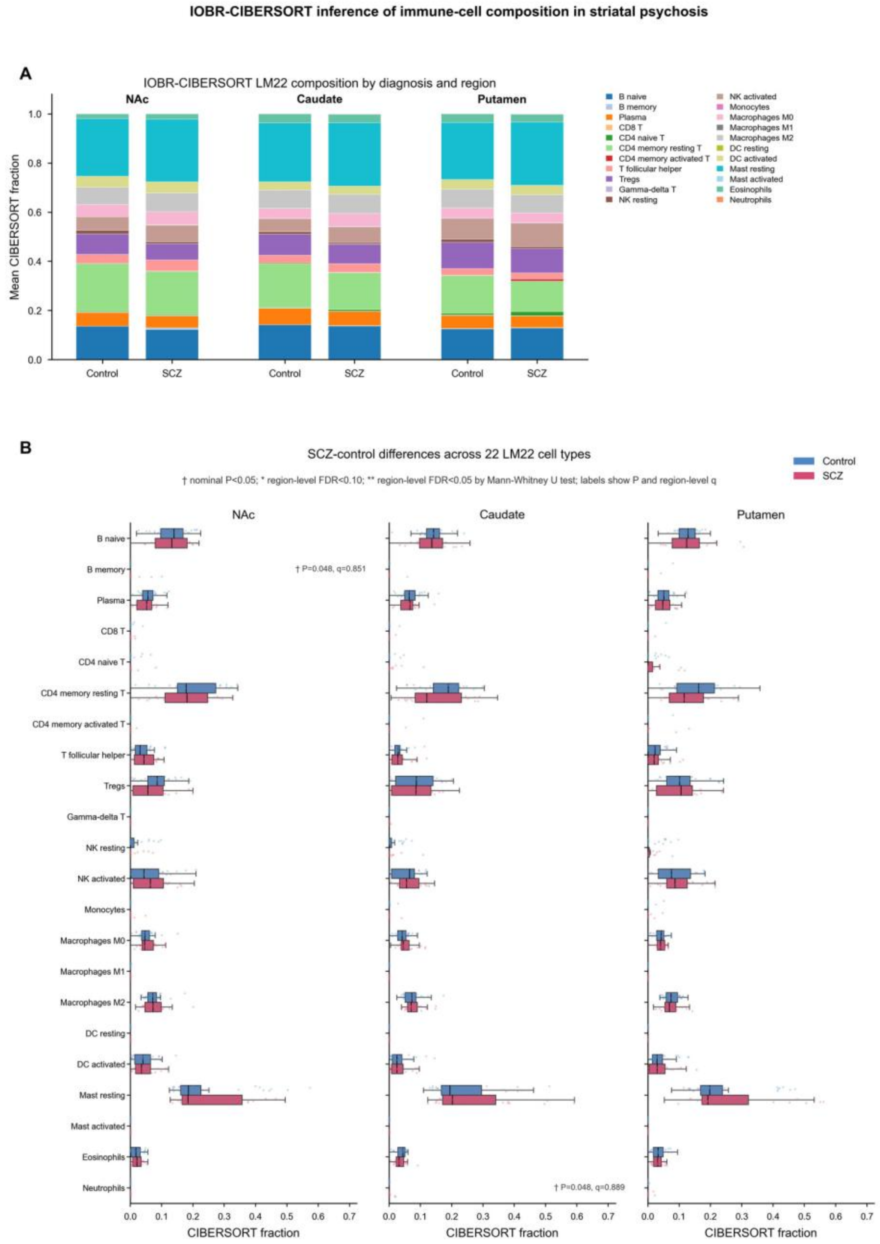

Figure S6. IOBR-CIBERSORT LM22 immune deconvolution (leukocyte-reference sensitivity analysis). (A) Mean relative CIBERSORT fractions for the 22 LM22 immune-cell states, stratified by diagnosis and brain region. Each stacked bar sums to one. (B) Horizontal boxplots with jittered sample-level fractions for all 22 LM22 states in NAc, caudate, and putamen. Boxes show median and interquartile range; whiskers extend to 1.5 times the interquartile range. Control and schizophrenia were compared by two-sided Mann-Whitney U tests. Labels report nominal P and region-level Benjamini-Hochberg q values. Dagger denotes nominal P < 0.05; one and two asterisks denote regional FDR < 0.10 and < 0.05, respectively. LM22 was developed for leukocyte mixtures; null results are not evidence against infiltration.

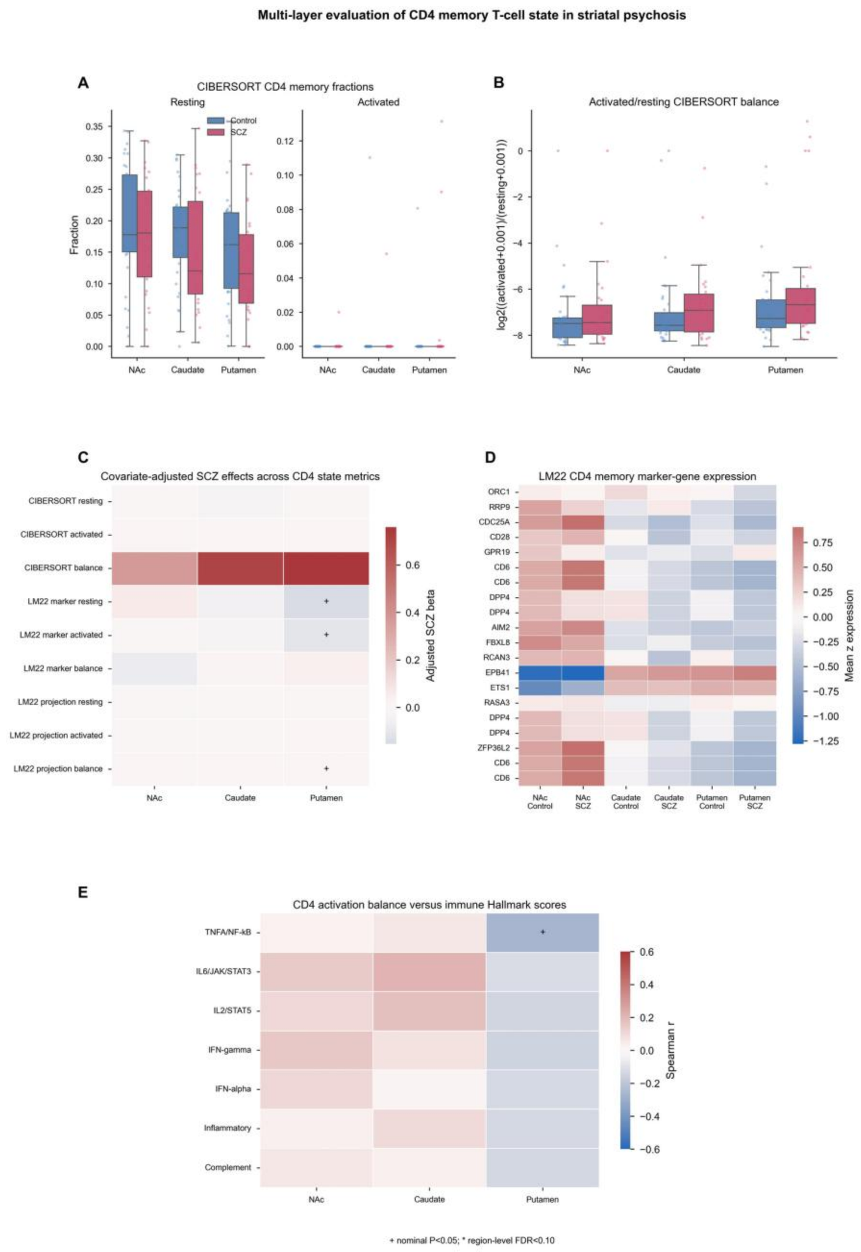

Figure S7. Complementary evaluation of CD4 memory T-cell state (leukocyte-reference sensitivity analysis). (A) CIBERSORT resting and activated CD4 memory fractions by diagnosis and brain region. (B) Activated-to-resting CIBERSORT balance, calculated as $\log_2[(\text{activated} + 0.001)/(\text{resting} + 0.001)]$. (C) Heatmap of covariate-adjusted schizophrenia coefficients for CIBERSORT fractions and balance, LM22-derived marker scores, and LM22 profile-projection scores. (D) Mean z-scored expression of the displayed LM22 CD4 memory marker genes by diagnosis-region group. (E) Spearman correlations between CD4 activation balance and immune Hallmark scores. Boxes show median and interquartile range with jittered samples. Plus signs denote nominal $P < 0.05$; asterisks denote region-level FDR < 0.10.

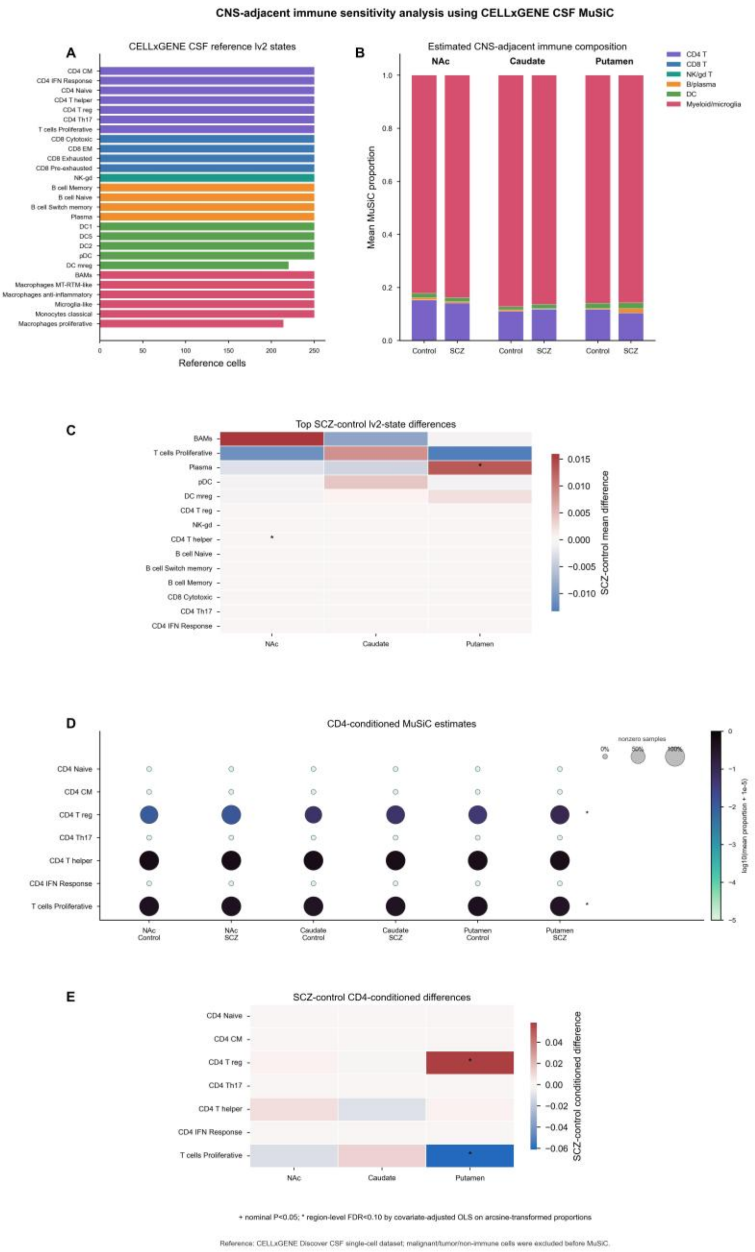

Figure S8. CNS-adjacent immune deconvolution using a CELLxGENE CSF single-cell reference (sensitivity analysis). (A) Reference-cell counts for 27 level-2 immune states after exclusion of malignant, tumor, and non-immune cells. (B) Mean MuSiC proportions grouped into broad immune classes by diagnosis and brain region. (C) Heatmap of schizophrenia-minus-control mean proportion differences for selected level-2 states from the general MuSiC model. (D) CD4-conditioned MuSiC estimates for seven CD4-related states; point size indicates the percentage of samples with nonzero estimates and color indicates $\log_{10}$(mean proportion + 10^{-5}). (E) Heatmap of schizophrenia-minus-control mean differences from the CD4-conditioned analysis. Covariate-adjusted tests used OLS models of arcsine-square-root-transformed proportions. Plus signs denote nominal $P < 0.05$; asterisks denote region-level FDR < 0.10.

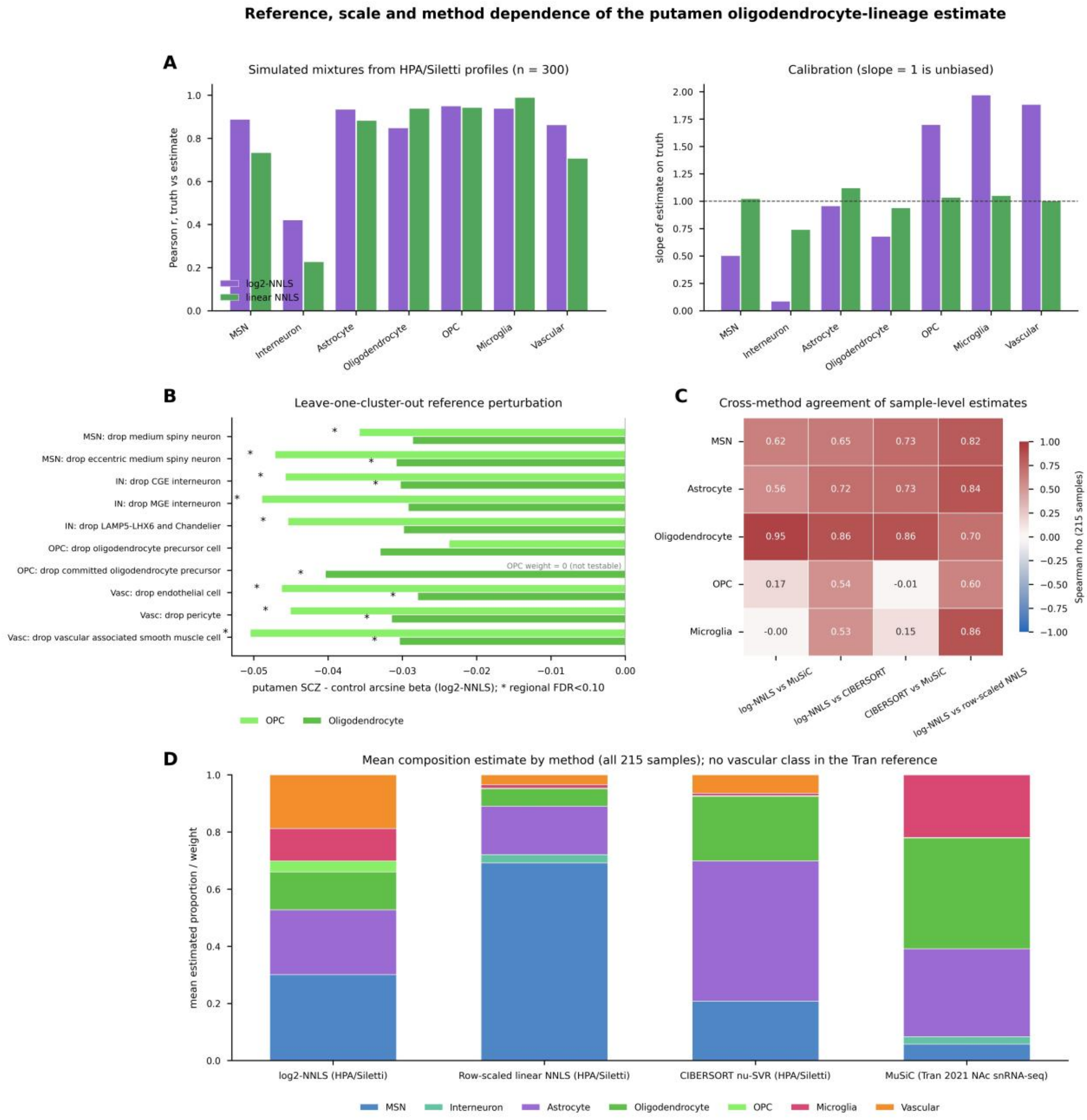

Figure S9. Reference, scale, and method dependence of composition estimates. (A) Recovery of simulated cell-class proportions (300 mixtures generated from HPA/Siletti cluster profiles, Poisson-sampled at 3×10^7 reads) by $\log_2$-scale and linear-scale NNLS: Pearson correlation between truth and estimate (left) and slope of estimate on truth (right). (B) Leave-one-cluster-out re-derivation of the marker reference: putamen schizophrenia-minus-control arcsine beta for OPC and oligodendrocyte weights after dropping each contributing cluster; asterisks denote regional FDR < 0.10. (C) Spearman correlation of sample-level estimates between estimators for five classes. (D) Mean composition estimate across all 215 samples for each estimator; the Tran et al. reference contains no vascular class.

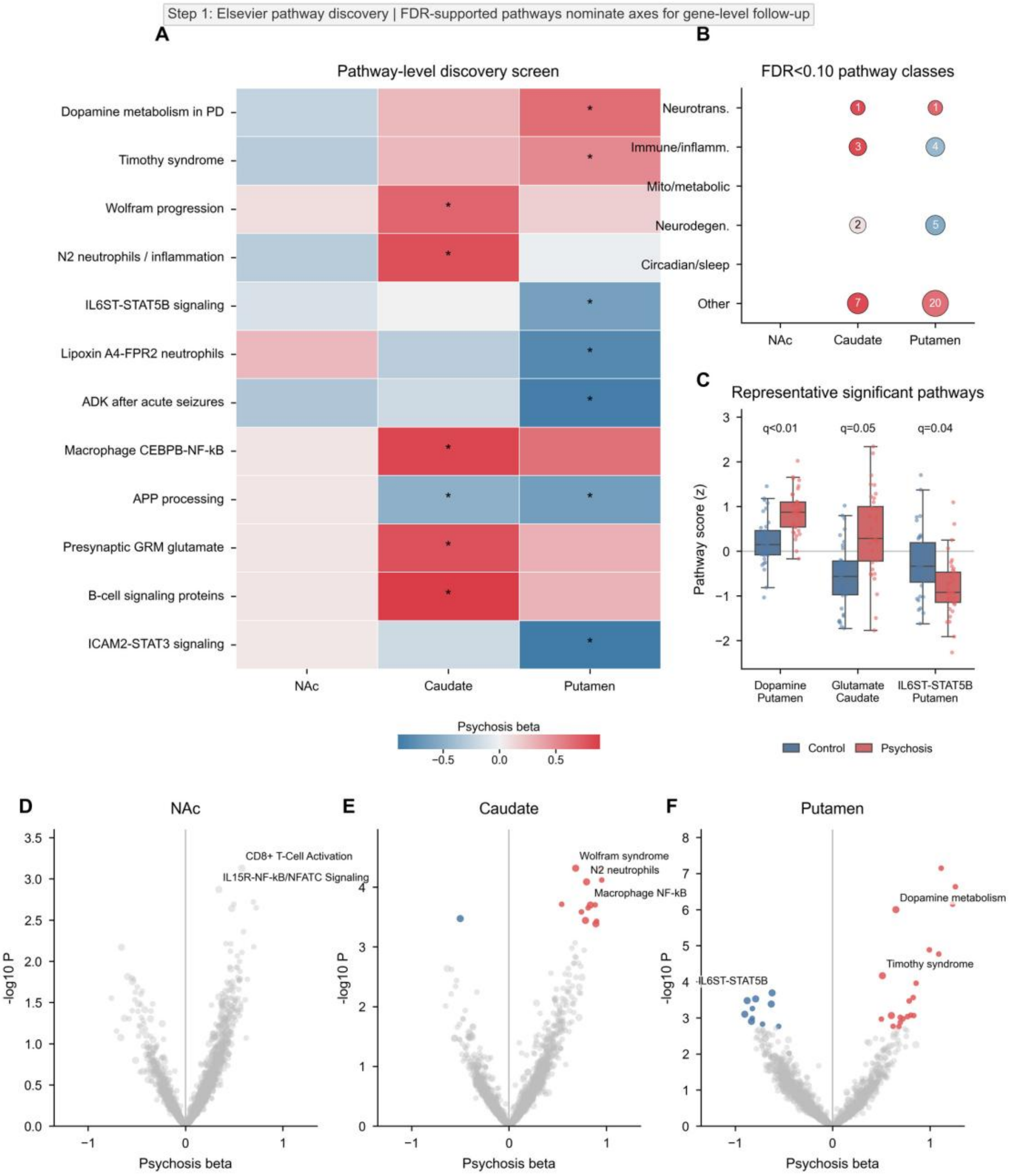

Figure S10. Secondary Elsevier pathway analysis of pooled psychosis. (A) Selected pathway coefficients across NAc, caudate, and putamen. (B) Counts and mean coefficients of pathways meeting FDR < 0.10 by class and region. (C) Sample-level scores for representative pathway-region pairs. (D) NAc volcano plot of pathway coefficient versus -log$_{10}$ nominal P value. (E) Corresponding caudate volcano plot. (F) Corresponding putamen volcano plot. Psychosis combines schizophrenia and bipolar disorder with psychosis. Asterisks denote regional FDR < 0.10.

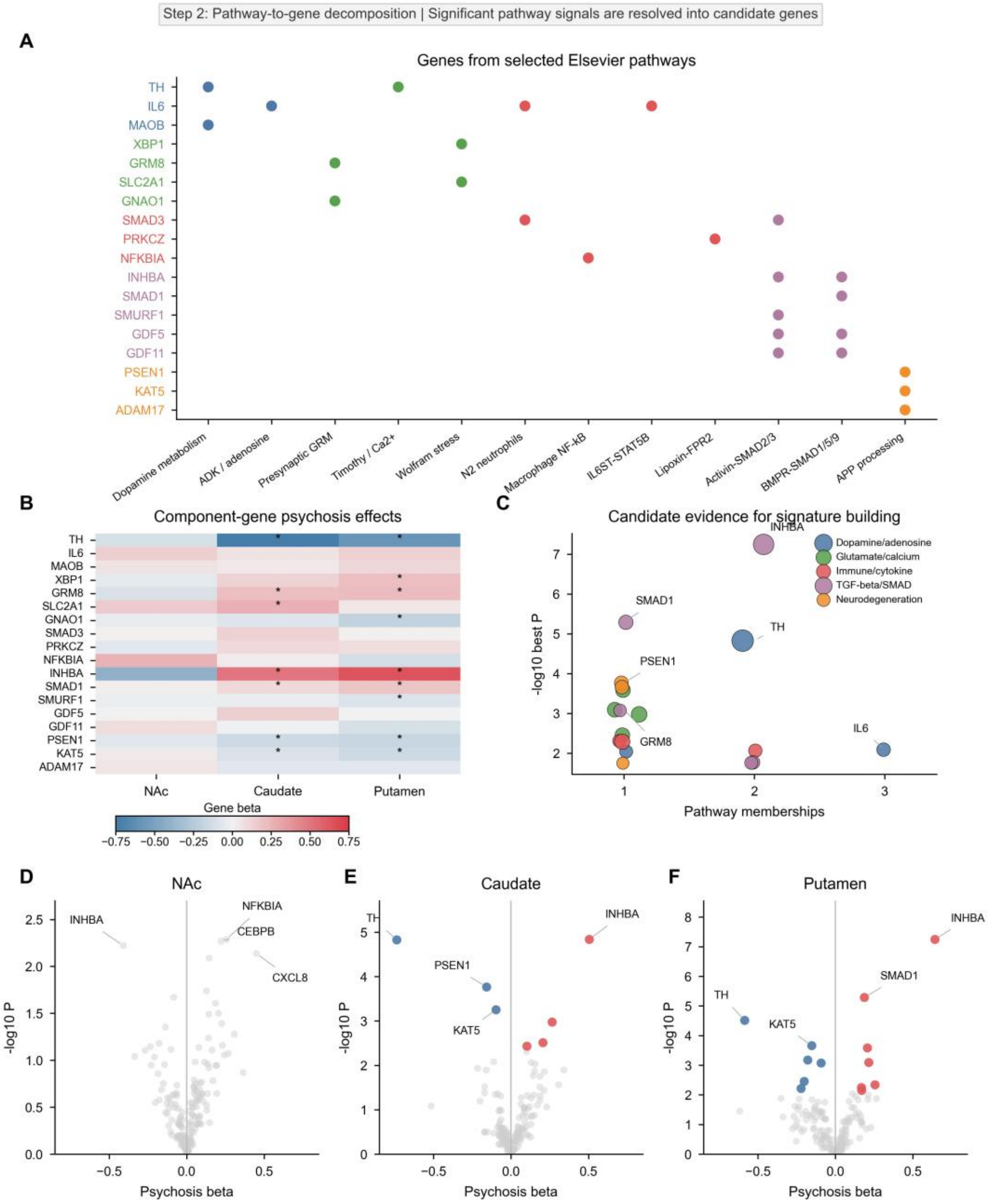

Figure S11. Candidate-gene analysis within selected Elsevier pathways. (A) Pathway-gene membership map. (B) Region-specific pooled-psychosis gene coefficients. (C) Candidate prioritization by selected-pathway membership count and -$\log_{10}$ minimum model P value. (D) NAc candidate-gene volcano plot. (E) Corresponding caudate volcano plot. (F) Corresponding putamen volcano plot. Highlighted points identify selected candidate genes; asterisks denote regional FDR support.

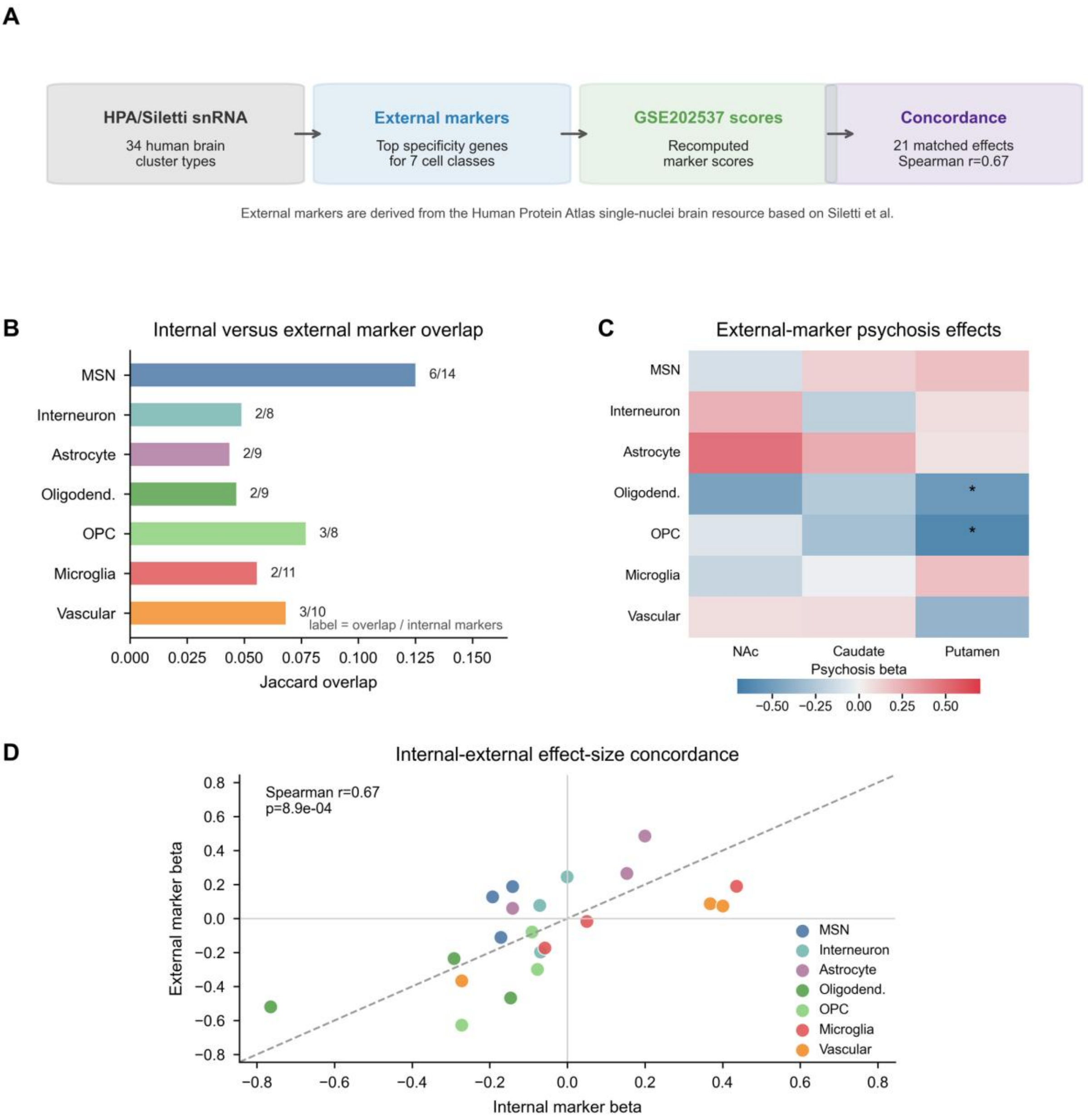

Figure S12. External HPA/Siletti marker cross-reference. (A) Workflow for deriving seven broad cell-class marker sets from 34 single-nucleus cluster types and rescoring them in GSE202537. (B) Jaccard overlap between internal and external marker sets; labels show overlap divided by internal-marker count. (C) External-marker pooled-psychosis coefficients across regions. (D) Concordance between matched internal and external marker-score coefficients across 21 class-region pairs. Asterisks denote regional FDR < 0.10; concordance was evaluated by Spearman correlation.
